# Development of Rapidly Dissolving Microneedles Integrated with Valsartan-Loaded Nanoliposomes for Transdermal Drug Delivery: In Vitro and Ex Vivo Evaluation

**DOI:** 10.3390/pharmaceutics17040483

**Published:** 2025-04-07

**Authors:** Ramsha Khalid, Syed Mahmood, Zarif Mohamed Sofian, Zamri Chik, Yi Ge

**Affiliations:** 1 Department of Pharmaceutical Technology, Faculty of Pharmacy, Universiti Malaya, Kuala Lumpur 50603, Malaysia; ramshaamaheen@gmail.com (R.K.); ms_zarif@um.edu.my (Z.M.S.); 2 Universiti Malaya-Research Centre for Biopharmaceuticals and Advanced Therapeutics (UBAT), Department of Pharmacology, Faculty of Medicine, Universiti Malaya, Kuala Lumpur 50603, Malaysia; zamrichik@ummc.edu.my; 3 Centre of Advanced Materials (CAM), Faculty of Engineering, Universiti Malaya, Kuala Lumpur 50603, Malaysia; 4 School of Pharmacy, Queen’s University Belfast, Belfast BT9 7BL, UK

**Keywords:** valsartan, rapidly dissolvable microneedle, liposomes, transdermal delivery, skin penetration, sustained drug release

## Abstract

**Background:** Hypertension (HTN) is recognized as a major risk factor for cardiovascular disease, chronic kidney disease, and peripheral artery disease. Valsartan (VAL), an angiotensin receptor blocker drug for hypertension, has been limited due to its poor solubility and poor absorption from the GIT, which leads to low oral bioavailability. **Objectives/Method:** In the present research, firstly, VAL-loaded nanoliposomes were formulated and optimized using the Box–Behnken design (BBD). Optimized VAL-nanoliposomes were physically characterized and their fate was examined by scanning and transmission microscopy, DSC, FTIR, XRD, and ex vivo studies using rat skin. In vitro studies using human keratinocyte (HaCaT) cells showed a decrease in cell viability as the liposome concentration increased. Secondly, the formulation of VAL-loaded nanoliposomes was integrated into dissolvable microneedles (DMNs) to deliver the VAL transdermally, crossing the skin barrier for better systemic delivery. **Results:** The optimized nanoliposomes showed a vesicle size of 150.23 (0.47) nm, a ZP of −23.37 (0.50) mV, and an EE% of 94.72 (0.44)%. The DMNs were fabricated using a ratio of biodegradable polymers, sodium alginate (SA), and hydroxypropyl methylcellulose (HPMC). The resulting VAL-LP-DMNs exhibited sharp pyramidal microneedles, adequate mechanical properties, effective skin insertion capability, and rapid dissolution of the microneedles in rat skin. In the ex vivo analysis, the transdermal flux of VAL was significantly (5.36 (0.39) μg/cm^2^/h) improved by VAL-LP-DMNs. The enhancement ratio of the VAL-LP-DMNs was 1.85. In conclusion, liposomes combined with DMNs have shown high potential and bright prospects as carriers for the transdermal delivery of VAL. **Conclusions:** These DMNs can be explored in studies focused on in vivo evaluations to confirm their safety, pharmacokinetics profile, and pharmacodynamic efficacy.

## 1. Introduction

Transdermal drug delivery systems (TDDSs) have emerged as a widely explored non-invasive method for delivering drugs through the skin, offering controlled and sustained drug release into the systemic circulation [1]. The efficiency of drug delivery through the transdermal route has been widely studied over the past three decades, with vesicle carriers, for example, transfersomes, liposomes, niosomes, ethosomes, and microneedles, potentially acting by bypassing the metabolic first-pass effect and allowing for sustained drug release over time [2,3,4,5,6]. Recent advancements in transdermal drug delivery have introduced innovative techniques, such as microneedles (MNs) integrated with nanoparticles, enhancing drug administration with increased bioavailability and reduced pain, enabling direct systemic absorption for improved efficacy [7]. The MN delivery system, a recent innovative platform, consists of an array of sub-millimeter-sized needles (up to 1500 μm in length) that are able to penetrate the viable epidermis of the skin, bypassing the outermost layer of the skin [8]. Several types of MN systems exist, including solid MNs, coated MNs, hollow MNs, and dissolvable MNs (DMNs) [9]. Among the different types, DMNs have gained increasing attention due to their biocompatibility, ability to create transient microchannels in the skin, and complete dissolution after drug delivery, eliminating biohazardous sharp waste. A recently reported study focused on overcoming the skin’s barrier properties through a DMN-based approach, integrating both physical and chemical enhancers to facilitate the efficient transdermal administration of benidipine for hypertension management [10]. Additionally, a flurbiprofen axetil-loaded DMN system has been shown to effectively deliver flurbiprofen, reducing pain and significantly decreasing swelling and inflammatory markers for arthritis treatment [11]. DMNs have demonstrated their effectiveness in delivering drugs for various conditions, such as psoriasis with methotrexate and diabetes with insulin [12,13]. One of two substances is often used to prepare DMNs: polymers or sugars. Polymers are essential in the formulation of DMNs, ensuring mechanical strength, stability, and controlled drug release. In MN-based systems, the choice of polymer influences the needle dissolution, penetration efficiency, and overall performance [14]. Sodium alginate (SA), a naturally occurring biodegradable polymer, is widely used in biomedical applications due to its biocompatibility, hydrophilicity, and ability to form stable gel matrices. Its hydroxyl and carboxyl functional groups enhance water solubility, making it a suitable candidate for DMN fabrication [15]. To further improve its mechanical properties, SA is often combined with other biodegradable polymers, such as chitosan and gelatin. Numerous studies have reported the combination of SA with various biodegradable polymers, e.g., high and medium hydroxypropyl cellulose (abbreviated as HPC-H and HPC-M), gelatin, and chitosan [16,17,18]. In the current study, SA was combined with HPMC to fabricate VAL-loaded DMNs, ensuring their structural integrity and efficient drug release upon skin insertion.

**Figure 1:**
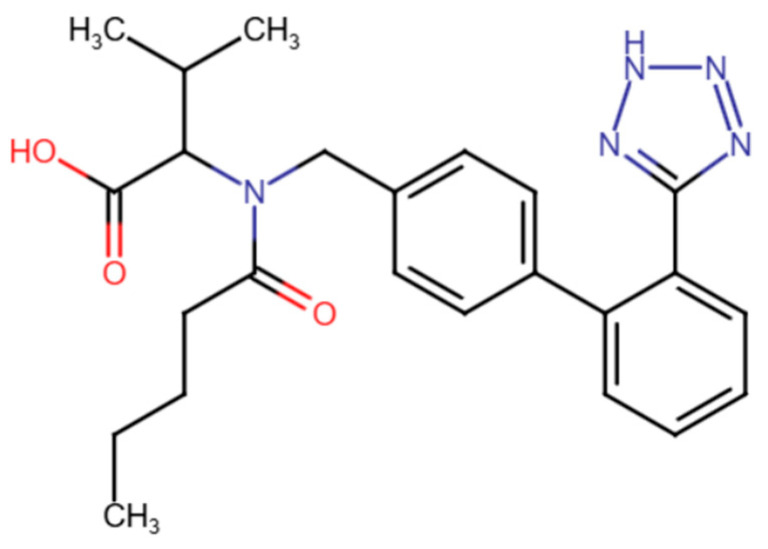
The molecular structure of valsartan.

Drug delivery via an MN-based transdermal route is ideally suited for chronic ailments, like hypertension, that require ongoing treatment. VAL, a highly selective angiotensin II type 1 receptor antagonist, is an orally active drug used to treat high blood pressure. VAL comes under BCS class II. The oral administration of VAL is limited by the first-pass effect and reduced gastrointestinal (GI) absorption, leading to a significant decline in both its maximum concentration (C_max_) and bioavailability [19]. Recent studies have developed valsartan (VAL)-based ethosomes and bilosomes to enhance their bioavailability for hypertension management while overcoming the limitations of oral administration [2,20]. For microneedle-based drug delivery, VAL possesses the ideal properties, including limited oral bioavailability (10–35%), a moderate elimination half-life (4–6 h), small molecular weight (435.5 g/mol), and increased lipid solubility (Log P = 1.499) [2]. The molecular structure of VAL consists of biphenyl and tetrazole moieties that contribute to its high lipophilicity, which makes it an ideal candidate for liposome formulations and subsequent dissolution in biodegradable MN matrices (Figure 1).

**Figure 2:**
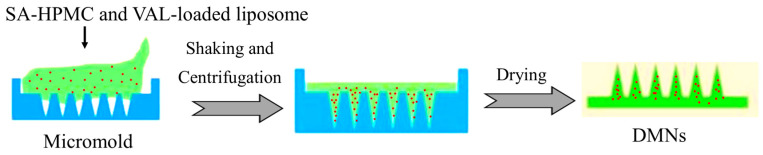
Schematic diagram of the integration of liposomes to DMNs.

The primary objective of this study is to explore the potential of DMNs integrated with VAL-liposomes as an innovative platform for enhancing the transdermal delivery of VAL and overcoming the limitations associated with its conventional delivery. In the current study, the liposomes were prepared using the thin-film hydration method, later optimized by response surface methodology (RSM), and characterized. The optimized liposomal formulation was then incorporated into SA-HPMC-based DMNs (Figure 2), which were characterized for their mechanical strength, insertion capability, and dissolution properties. The efficiency of optimized VAL-LP integrated into DMNs (VAL-LP-DMNs) in enhancing the transdermal delivery of VAL was investigated by ex vivo analysis. A significant improvement in VAL permeability was observed as compared to the VAL-DMNs due to the synergistic effect of both chemical and physical penetration enhancers— liposomes and DMNs—that create microchannels in the skin. This approach contributes to better drug permeation through the skin, offering a superior alternative to the traditional oral delivery method. This novel approach provides a promising strategy for improving therapeutic outcomes and patient compliance compared to existing formulations.

## 2. Materials and Methodology

### 2.1. Materials

Lipoid P100^®^ consists of phosphatidylcholine from soybean and was obtained from Lipoid GmbH (Ludwigshafen, Germany). Valsartan (active pharmaceutical ingredient) was purchased from Aladdin^®^ Biochemical Technology Co., Limited, Shanghai, China. Cholesterol was purchased from Diamond Sangon Biotech^®^ (Shanghai) Co., Ltd., Shanghai, China. Fluorescein isothiocyanate (FITC), as a tagging substance, was procured from Macklin Biochemical Co., Ltd. (Shanghai, China). Chloroform, methanol, absolute ethanol, and other HPLC-grade solvents such as acetonitrile and methanol were purchased from HmbG^®^ Chemicals Sdn Bhd, Selangor, Malaysia.

Sodium alginate (SA, AR 90% with M/G ratios 2:1), hydroxypropyl methylcellulose (HPMC, viscosity = 4000 mPa·s), polyvinyl alcohol (PVA, MW = 95%, 89,000–98,000), and polyvinyl pyrrolidine (PVP, MW = 58,000, K30) were purchased from Macklin Biochemical Co., Ltd., Shanghai, China. Polydimethylsiloxane (PDMS) molds for dissolving microneedles (6 × 6 arrays with needle heights of 1000 μm) were purchased from Micropoint Technologies Pte. Ltd. (Singapore). Nylon and cellulose acetate membranes of 0.45 μm were purchased from Thermo Scientific, Petaling Jaya, Malaysia. Ultrapure water was used during all the experimental work.

### 2.2. Preparation of Liposomes (VAL-LP)

The Valsartan-loaded liposome was prepared by the conventional thin-layer rotary evaporation method. Liposomes were prepared using lipoid P100 and cholesterol as a stabilizer (9:1 ratio with lipoid P100: cholesterol) [21]. In a round bottom flask, lipoid P100, VAL, and cholesterol were dissolved in a mixture of organic solvents, e.g., chloroform and methanol (2:1, *v*/*v*), to ensure a homogeneous mixture, and at 46 °C, the solvent was evaporated. After the solvent evaporated from the flask’s inner surface, the lipid film layer was allowed to hydrate for one hour in ultra-purified water from 45 °C to 46 °C and 200 rpm in an incubator shaker (Benchtop incubator shaker). During the hydration stage, the lipids become swollen and hydrated. Later, the vesicles underwent sonication using a probe sonicator (Qsonica sonicator, Newtown, CT, USA) to reduce their size further. The resulting small vesicles were then passed through a 0.45 μm pore size nylon membrane filter.

Fluorescent-tagged liposomes were prepared with the same procedure as above; 0.03% of dye was added to the lipid before thin-film formation.

### 2.3. Optimization of VAL-LP Formulation

The response surface methodology (RSM) design, such as the widely employed Box–Behnken design (BBD), is utilized for statistically optimizing formulations. This quadratic design is characterized by its rotatable or near-rotatable and non-dependent nature, with different treatment combinations positioned at the process space’s midpoints. Design-Expert^®^ (State-Ease Inc., Minneapolis, MN, USA) version 13.0 was used to optimize the VAL-LP formulation utilizing BBD. Three distinct factors are evaluated at three different levels in a BBD; the codes for low, medium, and high levels are −1, 0, and +1, respectively. The study considered three independent and dependent variables: The lipoid P100 amount (A), sonication time (B), and amount of VAL (C) were selected as independent variables for the BBD. The dependent variables for the BBD were the particle sizes of the vesicles (Y_1_), the zeta potential (ZP) (Y_2_), and the entrapment efficiency % (EE%) (Y_3_). The ranges for the chosen independent variables of the BBD were determined based on the findings of preliminary studies of our group and other relevant literature [10,22,23]. Table 1 displays the ranges of independent variables selected, including their three levels. Each formulation of VAL-loaded liposome was prepared with a volume of 10 mL. The experimental VAL-loaded liposome formulations, with different factor combinations containing five central points, were prepared using the BBD.

### 2.4. Particle Size (PS), PDI (Polydispersity Index), Zeta Potential (ZP), and Entrapment Efficiency (EE%)

The particle size (PS), PDI (polydispersity index), and zeta potential (ZP) of the VAL-LP formulations were determined using the dynamic light scattering (DLS) technique. A Malvern series zetasizer (Nanosizer, Malvern Instruments Ltd., Malvern, UK) at 25 °C and a scattering angle of 90° was used for the analysis. Before the measurements, approximately 0.1 mL of the sample was diluted in 1.9 mL of ultra-purified water. All measurements were taken in triplicate (n = 3).

The entrapment efficiency of the VAL-LP formulations was determined by an indirect method using centrifugation. This process involved centrifugation of the samples at 14,000 rpm for 30 min at 4 °C (relative centrifugal force (RCF) ≈ 9373× *g*) using a refrigerated microcentrifuge (Fresco™ 21, Benchtop Microcentrifuge, Thermo Fisher Scientific, Waltham, MA, USA) to isolate the obtained vesicles from the unencapsulated drug [24]. The resulting supernatant, containing the free drug molecule, was then analyzed using the HPLC technique. Each measurement was conducted three times and according to the following equation:

(1) $$EE\%=\frac{Q_{t}-Q_{s}}{Q_{s}}$$

where *Q_t_* represents the theoretical drug quantity added to each liposome formulation, and *Q_s_* denotes the drug quantity detected in the supernatant. Each measurement was conducted three times.

#### HPLC Method

An Agilent 1220 Series HPLC unit with a detector device (Agilent Technologies Inc., Santa Clara, CA, USA) was used to validate the valsartan analytical HPLC method. The analytical HPLC method, developed by Kachave et al., 2018, was employed for valsartan analysis with a modification in the mobile phase ratio [25]. Chromatographic analysis was performed on a Phenomenex C-18 column (particle size: 5 µm; column length and diameter: 150 mm × 4.68 mm) using an isocratic mobile phase of 0.01 N phosphate buffer (pH adjusted to 3.0) and acetonitrile (50:50). The flow rate was set to 1.0 mL/min, with detection at a 280 nm wavelength, and a 20 μL injection volume. Following the preparation of the mobile phase, the standard solutions were also prepared. A standard stock solution of 400 µg/mL was prepared by dissolving 40 mg of valsartan in 100 mL of the mobile phase, using it as a diluent. Serial dilutions were performed to obtain valsartan working solutions with different concentrations of 5, 10, 20, 30, 40, 50, 60, and 80 µg/mL. The standard calibration curve’s linearity for the VAL concentrations between 5 and 80 µg/mL was determined using a regression equation of Y = 5693.2X − 8905.2, with a correlation coefficient (R^2^) of 0.9998.

### 2.5. Differential Scanning Calorimetry (DSC), Fourier Transform Infrared Spectroscopy (FTIR), and X-Ray Diffraction (XRD) Analysis

Thermal analysis of pure VAL, lipoid P100, cholesterol, and the freeze-dried optimized VAL-LP formulation was conducted using a DSC (DSC 6000, Perkin Elmer, Waltham, MA, USA). An aluminum crucible was sealed and filled with the sample, which weighed approximately 2–5 mg [26]. FTIR spectroscopy was used to conduct a drug–carrier interaction study to determine the compatibility of the drug with the liposome. All samples were analyzed using a Fourier transform infrared spectrophotometer (Perkin Elmer Inc., Waltham, MA, USA) [27]. XRD analysis was used to examine the crystallinity of VAL in the optimized lyophilized VAL-LP formulation. An X-ray diffractometer (Malvern Panalytical, Malvern, UK) was used to record the diffraction peaks [28].

### 2.6. Surface Morphology and Size Analysis

The shape, size, and surface morphology of the VAL-LP were visualized by force emission scanning electron microscopy (FESEM), high-resolution transmission electron microscopy (HRTEM), and atomic force microscopy (AFM). The FESEM measurements were performed using a glass cover slip. A drop of VAL-LP suspension with 1:1 dilution with ultra-purified water was completely dried in an oven. After drying, it was coated with platinum and visualized by FESEM (JÉOL JSM-7800F, Akishima, Japan) [29]. HRTEM analysis was carried out after placing a drop of the VAL-LP sample on the copper film-coated grid. Negative staining was performed using 1% (*w*/*v*) phosphotungstic acid solution, followed by air drying for 30 min and visualization by the HRTEM instrument [Brand: JEOL, Model: JEM-2100F] [30,31]. AFM is an effective technique for examining the structural characteristics of lipid vesicles. This method is based on quickly scanning a sample placed on a mica surface using a nanoscale probe [32]. The AFM investigation was carried out using a Nasoscope V controller and AFM Catalyst (Bruker BioScope Catalyst, Billerica, MA, USA).

### 2.7. Confocal Laser Scanning Microscopy (CLSM)

CLSM technology was utilized to visualize the three-dimensional structure of the skin and the penetration of the liposomes in the skin [33]. The sample (VAL-LP tagged with FITC) underwent an eight-hour ex vivo skin-based permeation study using a Franz diffusion cell. After removing the skins, ultrapure water was used for a thorough washing. The treated skin area was vertically cut into slices that ranged in thickness from 8 to 12 µm. Then, a Nikon Ti2 Eclipse Confocal microscope (Melville, NY, USA) with a green filter (excitation peak at 495 nm and emission peak at 519 nm) was used to optically scan the treated skin.

### 2.8. Ex Vivo Analysis of Skin Permeation

Rat skin, provided as a gift by the Animal Experimental Unit (AEU), Faculty of Medicine, Universiti Malaya, was used as a permeation barrier. Before the experiment, the rat’s stratum corneum (SC), or outer layer of skin, was carefully shaved with a razor to remove hair without causing any damage to the skin surface. A modified Franz diffusion cell was used to measure the ex vivo permeation study of VAL from its respective drug-loaded liposome (VAL-LP) formulation. Skin from the abdomen of rats was employed as a permeation barrier. For VAL-LP, the receptor chamber was filled with a modified 60:40 (*v*/*v*) mixture of ethanol and air bubble-free PBS (pH: 7.4, simulated blood pH) [10,34]. The receptor medium was stirred at a pace of 100 rpm, and the temperature was maintained at 37 ± 2 °C. Based on the therapeutic transdermal daily dose (*Td*) of VAL, the dose of VAL employed for the ex vivo investigation was determined to avoid under-dosing using Equation (2) [35].

(2) $$Td=\frac{D_{0}\times F}{100}$$

where *F* represents the oral bioavailability percentage; *D*_0_ represents the oral drug dose. A sample (1 mL) was aliquoted from the diffusion cell’s receptor compartment at specific time intervals, e.g., 0.5, 1, 2, 4, 6, and 8 h, and later replenished with the same amount (1 mL) to maintain the sink condition.

### 2.9. MTT Assay

#### 2.9.1. Cell Line Preparation

The HaCaT cell line, a human keratinocyte line, was obtained from the American Type Culture Collection (ATCC^®^, Manassas, VA, USA). The cells were cultured in Dulbecco’s Modified Eagle Medium (DMEM) supplemented with 10% fetal bovine serum (FBS). The culture was maintained at 37 °C in a humidified atmosphere containing 5% CO_2_ in a CO_2_ incubator.

#### 2.9.2. MTT Assay

The MTT (3-[4,5-dimethylthiazol-2-yl]-2,5-diphenyltetrazolium bromide) assay is a widely used parameter to evaluate cellular metabolic activity, serving as a reliable indicator of cell viability, growth, and potential toxicity [36]. The % cell viability of liposomes loaded with (VAL-LP) or without drugs (empty liposomes, EL) towards HaCaT cells was evaluated by MTT assay. The optimized liposome formulation (VAL-LP) was diluted with DMEM. After that, 20 μL of the MTT solution, 5 mg/mL (20 μL; Ekear, Shanghai, China; cat. no. M0105), was added at the end of the incubation of the samples to the cells. After removing the supernatant, the solubilization of the purple formazan crystals using DMSO under constant shaking at 37 °C for 30 min was completed, and the cell viability of the sample was measured using an ELISA reader (model Infinite F50; Tecan, Männedorf, Switzerland) at a wavelength of 570 nm, with the untreated cells used as the control group. The IC_50_ value, defined as the drug concentration causing 50% growth inhibition of the cells, was also determined. The percentage cell viability was calculated using the formula given below:

(3) $$Cell\;viability=\frac{Absorbance\;sample\left(mean\right)}{Absrorbance\;control\;(mean)}\times 100$$

### 2.10. Preparation of Dissolving Microneedles (DMNs)

The DMNs were fabricated by using the micromold technique based on a combination of sodium alginate (SA, with an M/G ratio of 2:1; the molecular weight typically ranges between 100 kDa and 200 kDa, depending on the specific batch) with HPMC solutions (SA-HPMC-DMNs). Polydimethylsiloxane (PDMS) molds, measuring 6 × 6 and featuring needle heights of 1000 μm, were procured from Micropoint Technologies Pte. Ltd. (Singapore) for use in this study. Different combination ratios of SA-HPMC, 6:3, 8:4, and 10:5 %*w*/*v*, were prepared and degassed sufficiently for 30 min using an ultrasonic bath to remove the bubbles. The prepared SA-HPMC polymer solutions of different concentration ratios were placed onto the mold and shaken for 1 h at 150 rpm using an incubator shaker (Benchtop incubator shaker) to allow the polymer solution to fill the mold cavities. The filled mold was centrifuged (Hettich Universal-32 benchtop centrifuge; Andreas Hettich GmbH & Co. KG, Tuttlingen, Germany) for 60 min at 4000 rpm. Later, a backing layer of a polymer blend (10% concentration, with a PVP: PVA ratio of 4:6 %*w*/*v*) was cast onto the mold. The mold cast with a backing layer was kept in a drying oven (WTC Binder FD53 oven; Binder GmbH, Tuttlingen, Germany) for at least 12 h at 30 °C. The DMN array sheet was gently removed from the micromold once it was completely dried. Lastly, the desiccator was used to store the prepared DMNs. This study used three different concentration ratios of SA-HPMC (6:3, 8:4, and 10:5 %*w*/*v*) to prepare dissolvable microneedles (SA-HPMC-DMNs). The prepared DMNs were characterized by their morphology (shape), adequate mechanical strength, suitable insertion capability, and dissolution in rat skin. The selection criterion for the optimal SA-HPMC concentration was sharp pyramid needles, adequate mechanical properties, insertion ability, and rapid dissolution.

#### 2.10.1. Integration of Optimized VAL-LP Formulations into SA-HPMC-DMNs (VAL-LP-DMNs)

The optimized VAL-loaded liposome formulation was mixed into a 10:5 %*w*/*v* concentration of SA-HPMC to fabricate DMNs using the micromold technique (Figure 2). The final optimized microneedle matrix polymer solution was prepared by mixing the VAL-LP formulation with an equivalent amount of polymer 20:10 %*w*/*v* solution in a 1:1 ratio, resulting in a 10:5 %*w*/*v* SA-HPMC concentration. These prepared polymer solutions underwent degassing for 30 min to eliminate any air bubbles before being poured into the PDMS micromold cavities. The molds were then placed in an incubator shaker for 60 min, centrifuged for 1 h at 4000 rpm, and later dried for 12 h at 30 °C in an oven. Once completely dried, the dissolvable microneedle array was then gently peeled off from the PDMS mold. As a control sample, pure VAL-loaded DMNs were fabricated using the same micromold method, but instead of optimized liposome formulations, a hydro-ethanolic solution of VAL was mixed with a polymer solution. The prepared VAL-LP-DMNs were subsequently characterized for their morphology, mechanical strength, insertion capability, and dissolution of microneedles into rat skin [10].

#### 2.10.2. Morphology of DMNs

The digital microscope (Olympus BX60; Olympus, Tokyo, Japan) was used to observe the morphology of the DMNs. The DMNs (VAL-LP-DMNs) were coated with gold and evaluated using the scanning electron microscope (SEM; Quanta FEG 650 SEM chamber; Thermo Fisher Scientific, Waltham, MA, USA) to determine the shape and dimensions of the DMNs.

#### 2.10.3. Mechanical Strength of DMNs

The mechanical strength test of the DMNs was performed in the compression mode utilizing a CT3 texture analyzer (Brookfield Laboratories, Middleboro, MA, USA) [37]. The DMNs were affixed to a texture analyzer probe and pressed vertically onto a flat aluminum surface. The test parameters, simulating average human application force, included a 0.5 mm/sec test speed, 32 N force, and a 30 s hold time. For context, the maximum force typically applied by humans when using DMNs is 32 N [38]. An inverted optical microscope (Olympus BX60) was used to examine the DMNs before and after compression testing. The compression percentage was calculated by comparing the initial height of the DMNs and the post-compression heights of the DMNs (dividing the initial height by the diffraction height of the DMNs) [39].

#### 2.10.4. Insertion Capability of DMNs

A texture analyzer (TA) was used for the in vitro studies to analyze the DMNs’ insertion capability. DMNs were pressed against Parafilm M^®^ (Bemis Company Inc., Soignies, Belgium), a semi-transparent and thermoplastic sheet with a flexible nature composed of olefin-type polymer. The parafilm was folded into four layers for DMN insertion analysis [40]. The thicknesses of the 1st, 2nd, 3rd, and 4th layers of parafilm were 125 μm, 250 μm, 375 μm, and 500 μm, respectively. The movable probe of TA, with attached DMN arrays, was set at 0.5 mm/s for 15 s with 32 N force. An ex vivo insertion capability of optimized DMNs (VAL-LP-DMNs) was evaluated using prepared rat abdominal skin. The insertion ratio was determined by dividing the total number of holes created post-insertion by the total needles in the array [41].

#### 2.10.5. Dissolution of DMNs Using Rat Skin

The time required for the dissolution of DMNs was measured by pressing DMNs to the skin using a texture analyzer. The dissolving ability of the DMNs was determined at specified time intervals of 2, 4, 6, and 8 min. The DMNs were morphologically assessed at specified time intervals using an inverted optical microscope (Olympus BX60) to estimate the height of the DMNs [10,42].

#### 2.10.6. SEM and CSLM Analyses of Rat Skin Punctured with VAL-LP-DMNs

To assess the mechanical interaction between VAL-LP-DMNs and skin, DMNs penetrated the skin, and the resulting puncture sites were analyzed using SEM. This analysis aimed to analyze the penetration sites (holes created onto the skin) and insertion depth of the microneedles, providing insights into the effectiveness of the needle–skin interaction for drug delivery [43]. The skin area treated with microneedles was isolated, dried in an oven, and mounted on aluminum-based stubs using double-sided adhesive tape of carbon. The treated skin samples were then gold-coated for the SEM analysis. Likewise, the FITC-tagged VAL-LP-DMNs penetrated the skin. The treated skin area was vertically cut into slices and placed onto a glass slide along the z-direction. A Nikon Ti2 Eclipse Confocal microscope (Melville, NY, USA) with a green filter (with an excitation peak at 495 nm and emission peak at 519 nm) was used to optically scan the DMN-treated skin.

#### 2.10.7. Drug Content Determination in DMNs

The drug content in the needle area of the VAL-LP-DMNs was evaluated. The microneedles were dissolved in a hydro-ethanolic solution and agitated for 20 min with a vortex mixer until completely disrupted. Ultrapure water was used as the solvent because it can dissolve the SA-HPMC microneedle matrix [44]. However, since VAL and liposomes are water-insoluble, ethanol was added to help dissolve the drug and lipids as well. Subsequently, the solutions were centrifuged at 4000 rpm for 20 min. Following their separation, the supernatants were filtered through a 0.45 µm nylon-based filter membrane and later subjected to validated HPLC analysis.

#### 2.10.8. Ex Vivo (Skin Permeation and Deposition) Analysis of VAL-LP-DMNs

Rat skin was prepared as discussed in Section 2.10. The ex vivo investigation and permeation for VAL-LP-DMNs and VAL-DMNs (control group) were conducted using a custom-built Franz diffusion cell. For the experiment, the optimized VAL-LP-DMNs were applied to the skin. The skin was positioned on the receptor compartment of the diffusion cell after DMN insertion, with the stratum corneum facing the donor compartment. To secure the DMN arrays, high-adhesion double-sided medical tape was applied. The receptor compartment contained a release medium of VAL (modified ethanol and PBS with a 7.4 pH and a 60:40 *v*/*v* ratio), was stirred at 100 rpm, and maintained a constant temperature of 37 ± 2 °C. At predetermined intervals of 0.5, 1, 2, 4, 6, and 8 h, the sample of 1 mL was aliquoted with an immediate replenishment of fresh medium each time. The HPLC method was utilized to analyze the permeated samples. The steady-state transdermal flux (J_ss_) (μg/cm^2^/h) is derived from the slope of the standard linearity graph [45]. The enhancement ratio (ER) was calculated using Equation (4) to compare the sample with the control [46].

(4) $$Enhancement\;ratio\;(ER)=\frac{Steady\;state\;flux\;of\;formulation}{Steady\;state\;flux\;of\;control}$$

### 2.11. Statistical Analysis

Statistical analysis was performed using one-way analysis of variance (ANOVA). A *p*-value < 0.05 was considered statistically significant.

## 3. Results and Discussion

### 3.1. Optimization of VAL-LP Formulations

The basic components of the liposome formulations include the drug (VAL), phospholipid (lipoid P100; Lipoid GmbH, Ludwigshafen, Germany), and cholesterol as a stabilizer. To optimize the VAL-LP formulations, the effects of each selected independent variable for the BBD, for example, lipoid P100 (A), sonication duration (B), and the amount of drug (VAL) (C), at three levels (−1, 0, +1) were studied. These were measured against dependent variables like the size of the vesicles (Y_1_), ZP (Y_2_), and EE% (Y_3_). The VAL-loaded liposome formulations were prepared using the BBD in Design Expert^®^, Version 13, comprising 17 experimental liposome formulations with five central points, as illustrated in Table 2.

#### 3.1.1. Response 1: Impact of Independent Variables on VAL-LP Vesicle Size

The BBD-derived fitted mathematical polynomial Equation (5), which illustrates the impact of independent variables on vesicle size (Y_1_), is expressed as follows:

Particle size = 248.52 + 65.8663 A − 3.08787 B + 10.3616 C − 11.875 AB + 20.9925 AC − 17.5657 BC − 60.6266 A^2^ + 11.6316 B^2^ + 12.5491 C^2^ (5)

where the terms mentioned in the above equation, A, B, and C, represent the coded values for the lipoid P100 amount (mg), sonication duration (s), and VAL amount (mg), respectively. Regression analysis shows a synergistic effect, indicating that a positive value corresponds to a direct link with optimization, whilst a negative value displays an antagonistic effect, denoting an inverse association with optimization [23]. The significance of the model was evaluated using ANOVA; the low *p*-value (<0.05) and high F-value of the proposed model indicate its significance [30]. The developed model revealed that the terms mentioned were statistically significant (*p* < 0.05), except for some terms (highlighted in bold) (Table 3).

The observed VAL-LP vesicle size ranged from 127.4 (1.01) to 295.4 (0.31) nm. The liposome vesicle sizes were less than 300 nm, which is ideal for successful transdermal administration [47]. A study by Nava et al. reported that the optimal liposome size for transdermal drug delivery is up to 300 nm, facilitating deeper skin penetration and enhanced permeation [48]. VAL-LP’s vesicle size increased with the lipoid P100 concentrations, while the decreased concentration of phospholipid resulted in smaller particles, indicating suitability for transdermal drug delivery. Shaker et al. observed that increasing the phospholipid concentration in the aqueous phase led to a larger mean size of particles [49]. Taghizadeh et al. reported similar results that the particle size of liposomes decreases with a decreasing phospholipid concentration [50]. These findings align with previous studies and may be attributed to the formation of multilamellar vesicles when the lipid concentration increased, leading to a bigger vesicle size [51]. The sonication time (B) and the amount of VAL (C) itself showed no significant effect on particle size. This finding was consistent with the reported study showing no effect of the sonication time and amount of VAL on vesicle size. It was reported that the drug concentration had no significant effect on particle size. Instead, parameters such as lipid concentration and lipid type had a greater influence on particle size [2]. The contour and 3D response surface plots are used to depict the combination of two independent factors’ impact on the VAL-LP vesicle size (nm), as illustrated in Figure S1. Vesicle size and PDI are crucial factors in the analysis of nanoparticles because they have an impact on the stability, safety, and efficacy of nanoparticles as drug delivery vehicles. It is essential to understand the vesicle distribution to quantify the homogeneity of the formulation. The PDI is a measure of size distribution. The PDI of each liposome formulation was less than 0.3, as shown in Table S1. When liposome formulation is used for drug delivery, a PDI of 0.3 or lower is considered suitable, indicating a homogeneous or uniform population of phospholipid vesicles [52].

#### 3.1.2. Response 2: Impact of Independent Variables on VAL-LP Zeta Potential

The BBD-generated mathematical Equation (6), which showed the impact of the independent variables on ZP (Y_2_), is expressed as follows:

Zeta potential = −23.36 + 2.13334 A + 0.320837 B + 0.3875 C − 0.333325 AB + 1.75 AC + 0.675 BC + 1.40084 A^2^ + 2.37584 B^2^ + 0.659162 C^2^ (6)

where the terms mentioned in the above equation, A, B, and C, represent the coded values for the lipoid P100 amount (mg), sonication duration (s), and VAL amount (mg), respectively. The model is significant, as shown in Table 4, and a non-significant lack of fit, implying that the developed model is fit.

The zeta potential (ZP), which measures the net charge of the particles, is useful in predicting the stability of the system by determining the electrostatic repulsion between dispersed particles with similar charges [53]. The main cause of the negative charge in liposome formulations containing lipoid P100 (phosphatidylcholine) and cholesterol exposes the negatively charged phosphate groups on the liposome surface. Even though phosphatidylcholine (PC) is a molecule possessing both positive and negative charges, known as a zwitterion, it possesses phosphate groups that tend to deprotonate at neutral or physiological pHs, which adds a net negative surface charge to the liposomes [54]. All formulations of VAL-LP exhibited negative zeta potential values, indicating the stability of the liposomes. To stabilize the dispersed systems, a ZP of ±20 mV is considered desirable to stabilize the liposomes and protect against their aggregation [54,55]. The lipoid P100 concentration considerably affected the zeta potential. The ZP was not significantly affected by valsartan (C) itself; however, the ZP seems to be impacted by valsartan (C) in combination with specific lipoid P100 concentrations (A). The ZP increased as the lipoid P100 concentration increased from 150 to 225 mg, as shown in the response surface plot and contour plots (Figure S2). Zeta potential values above +30 and below −30 mV are considered desirable for the stability of the formulations of lipid vesicles. When electrostatic and steric stabilization are coupled, a zeta potential of at least ± 20 mV is preferred [56]. All formulations showed nearly similar zeta potentials from −20.2 (0.75) to −25.9 (0.75) mV except for VAL-LP D4, D5, D8, and D14. This study’s outcome aligns with previous research findings, confirming that a value of ZP ± 20 mV or more than this value is considered acceptable for the stability of liposomes [57,58].

#### 3.1.3. Response 3: Impact of Independent Variables on VAL-LP Entrapment Efficiency (EE%)

The polynomial equation for the impact of independent factors on EE% (Y_3_) that was obtained from the BBD is as follows (Equation (7)):

EE% = 97.4298 + 4.65305 A − 0.645612 B − 3.34659 C + 0.564375 AB + 2.24042 AC − 0.72685 BC − 3.55654 A^2^ − 0.62886 B^2^ − 0.32886 C^2^ (7)

where the terms mentioned in the above equation, A, B, and C, represent the coded values for the lipoid P100 amount (mg), sonication duration (s), and VAL amount (mg), respectively. The model is significant (*p*-value < 0.05) and has a non-significant lack of fit, implying that the developed model is fit (as shown in Table 5).

As seen in Figure S3, various plots, 3D response surfaces, and 2D contours demonstrate the impact of the model-independent variables on the EE% of VAL-LP. The percentage of the entire drug that is integrated into the liposome is known as the EE%. Enhancement of the EE% was seen upon increasing the lipoid P100 concentration from 150 to 225 mg. Since VAL is a highly lipophilic drug that readily disperses and traps into the lipid phase, this may be explained by the total drug dispersion in lipoid P100. Additionally, multilamellar vesicle formation with increased drug entrapment within a lipid core with a high lipid content may have contributed to this outcome [59]. Similar findings were reported, showing that an increased lipid concentration led to a higher medium viscosity and accelerated lipid vesicle solidification, blocking diffusion of the drug into the exterior phase [22,60]. However, when the amount of VAL is increased from 22.5 to 30 mg, the EE% decreases, which could be owing to excessive leaking of the drug from the vesicular structure of the liposome. According to a reported study, drug entrapment occurs in both compartments of the vesicles: the bilayers and aqueous. When these compartments become saturated with drugs, the entrapping capability of the vesicles is limited [22,61]. Some studies reported that higher VAL concentrations lead to a decreased EE % due to lipid bilayer saturation. As the VAL concentration increases, the liposomal membrane becomes saturated, reducing drug entrapment efficiency. At higher doses, the drug disrupts lipid packing, reducing the liposome’s structural integrity and, as a result, decreasing entrapment efficiency [22]. Phospholipids with long fatty acid chains enhance vesicle membrane stability, preventing drug expulsion from the vesicles and resulting in improved drug efficiency [62]. The EE% varies due to many reasons; for instance, an increase in lipid concentration resulted in a higher EE%. The EE% is reduced as the VAL concentration exceeds specified levels due to the leakage of the drug. This conclusion is consistent with prior studies [60,63].

#### 3.1.4. Optimization of VAL-LP

The platform for optimization of a formulation based on the desirability function concept is offered by Design Expert, Version 13, software. This approach involves using polynomial equations to establish the relationship between independent and dependent variables [64]. An optimized formulation was referred to as the combination of lipoid P100 (161 mg), a sonication period (107 s), and VAL (18 mg), as shown in Table 6. The optimized VAL-LP formulation showed a PDI of 0.122 (0.02). The percentage drug loading of the optimized VAL-LP was 9.52 (0.44) %.

#### 3.1.5. Differential Scanning Calorimetry (DSC)

**Figure 3:**
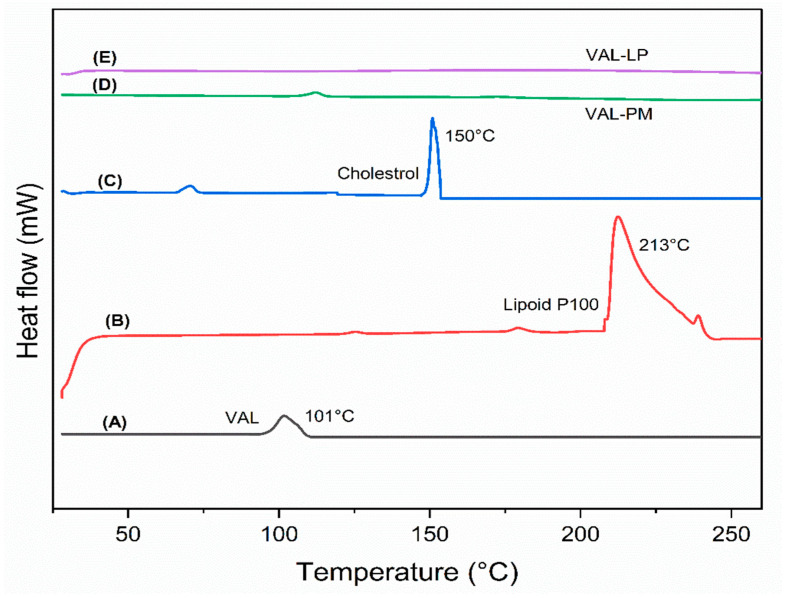
The DSC graph: (**A**) raw valsartan (VAL), (**B**) lipoid P100, (**C**) cholesterol, (**D**) physical mixture (PM) of VAL, lipoid P100, and cholesterol, and (**E**) the optimized VAL-LP formulation. (same quantity of all samples was used).

A DSC analysis was performed to observe the phase behavior of the liposomes and to investigate the thermal interaction among the drugs and excipients used in liposome formulations [65,66]. The thermograms of raw VAL, lipoid P100, cholesterol, and optimized liposomes (VAL-LP) are presented in Figure 3. The melting drug peak disappeared in the VAL-loaded liposome formulation. The absence of the melting drug endothermic peak in the VAL-LP formulation indicated the transformation of VAL into being less crystalline, becoming more amorphous, and gaining successful entrapment within liposomes [67]. Some reported studies, including thymoquinone-loaded liposome and Cordyceps sinensis-loaded liposome, also noted the absence of the drug’s thermal peak when integrated into liposome preparations [68,69].

#### 3.1.6. Fourier Transform Infrared Spectroscopy (FTIR)

**Figure 4:**
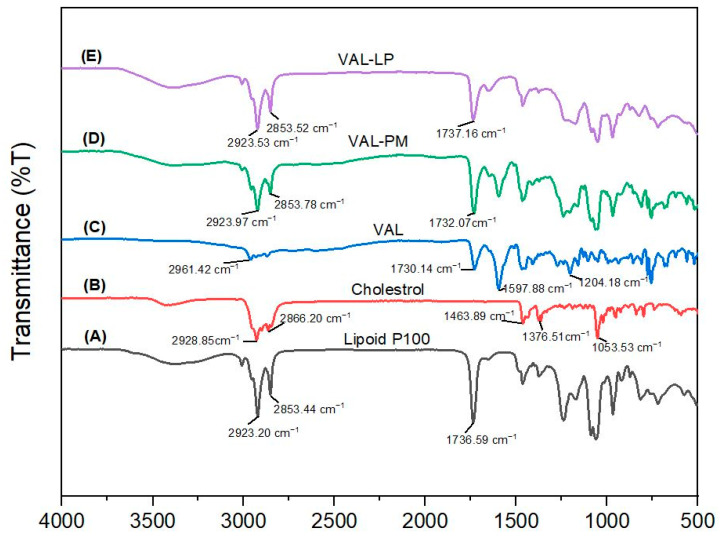
The FTIR spectrum: (**A**) lipoid P100, (**B**) cholesterol, (**C**) pure valsartan (VAL), (**D**) physical mixture (PM) of VAL, lipoid P100 and cholesterol, and (**E**) optimized VAL-LP formulation.

The potential interactions between VAL and the excipients used in the drug-loaded liposome formulations were investigated using FTIR spectroscopy. Figure 4 shows the FTIR spectra of all samples. The FTIR spectra of the optimized VAL-LP show peaks overlapping, and some of the drug (VAL) peaks disappeared, confirming the complexation behavior of VAL and lipoid P100. Drug entrapment in lipid vesicles (liposome) caused shifting, overlapping, or disappearance of the peak in optimal liposome formulations. The FTIR spectra of lyophilized VAL-LP demonstrated a drug–lipid interaction through the appearance of lipid peaks at 2923 cm^−1^, 2853 cm^−1,^ and 1737 cm^−1^ for both formulations, indicating drug–lipid complex formation [70].

#### 3.1.7. X-Ray Diffraction (XRD)

**Figure 5:**
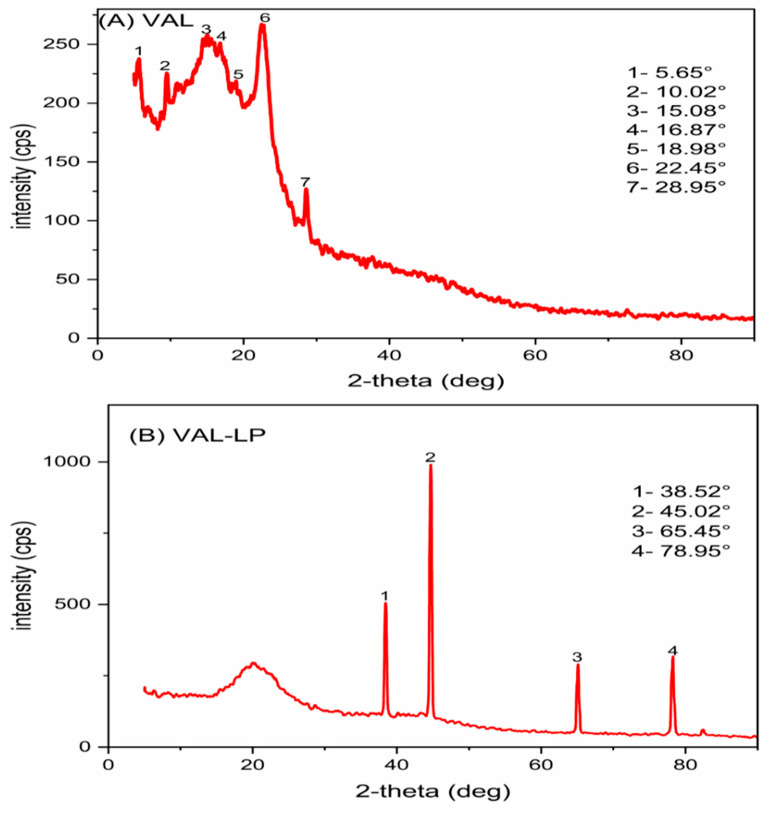
The XRD pattern: (**A**) pure valsartan (VAL) and (**B**) the optimized VAL-LP formulation.

XRD analysis was conducted for VAL, as well as optimized VAL-LP, as indicated in Figure 5, to examine the crystallinity changes and transformations during the liposome preparation. The diffraction spectrum of pure valsartan revealed a mixed nature of both crystalline and amorphous structures, as indicated by the hump-shaped peaks [71,72]. The peaks in the optimized liposome formulation (VAL-LP) were suppressed, and just a few peaks were visible. The drug incorporated inside the liposome exhibits decreased crystalline behavior, a sign of successful incorporation into the liposome’s lipid bilayer. The observed peaks likely represent the residual crystallinity, although the general decrease in peak strength is indicative of the encapsulated drug’s amorphous nature. Similar outcomes, including diminished peak intensity and state transformation, have been reported by many researchers [73,74].

#### 3.1.8. Field Emission Scanning Electron Microscopy (FESEM), High-Resolution Transmission Electron Microscopy (HRTEM), and Atomic Force Microscopy (AFM)

**Figure 6:**
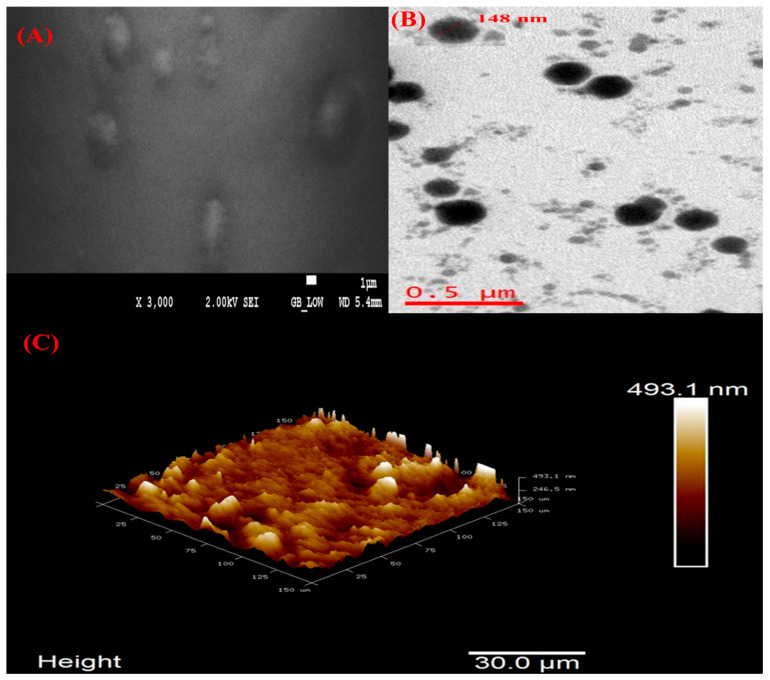
(**A**) FESEM image of optimized VAL-LP with 2 kV and ×3000 magnification; (**B**) HRTEM images of optimized VAL-LP with magnification 6 kx; and (**C**) AFM 3D image of optimized VAL-LP.

The morphology of optimized VAL-LP was examined using FESEM analysis, as shown in Figure 6A. The optimized liposome formulations exhibited a uniform spherical morphology. The HRTEM analysis showed that spherical and round-shaped vesicles predominated in the VAL-LP formulation (Figure 6B). Notably, the optimized liposome formulations show no signs of vesicle breaking or cracking. AFM provides a clear picture of the particle topology [30]. The AFM scan of the optimized VAL-LP is shown in Figure 6C. The distribution of particles for each formulation is seen in the height 3D image. It has been demonstrated that particles produced under proper substrate conditions and dilution provide an improved image and topology [75].

#### 3.1.9. Confocal Laser Scanning Microscopy (CSLM)

**Figure 7:**
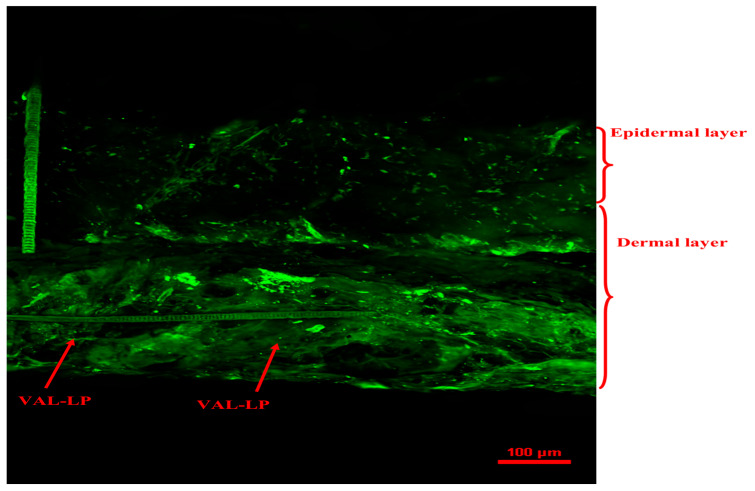
CLSM image of optimized VAL-LP penetration across rat skin after the 8th h of the ex vivo study.

CSLM microscopic analysis was conducted to assess the penetration of drug-loaded liposomes. The CLSM study examined the extent of drug penetration to deeper layers of the skin by the optimized VAL-LP. A fluorescent picture of the optimized VAL-LP is displayed in Figure 7. Image J software (version 1.54) was used to analyze the CLSM image that displayed the dye penetration depth and intensity; the intensity of VAL-LP was 587.03 (12.98), and the penetration depth was 183.47 (15.33) µm. The CSLM image of optimized VAL-LP revealed uniform fluorescence distribution throughout the layers of skin.

#### 3.1.10. Ex Vivo Study of VAL-LP

**Figure 8:**
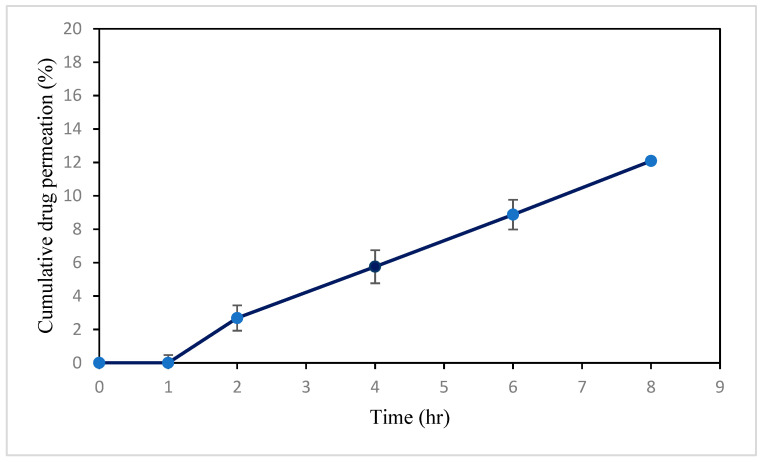
Image showing cumulative percentage drug release of optimized VAL-LP across rat skin during 8 h interval.

Ex vivo permeation analysis demonstrates absorbable drug levels; they provide important insights into the in vivo behavior of formulations [76]. The skin penetration in this study was probably made possible by the small particle size of optimized valsartan-loaded formulations and drug-encapsulating capacity. The transdermal flux (J_ss_) of the VAL-LP formulation was 9.53 (0.08) μg/cm^2^/h, indicating drug permeation through the skin. Figure 8 shows the percentage cumulative drug concentration graph over 8 h intervals.

#### 3.1.11. MTT Assay

**Figure 9:**
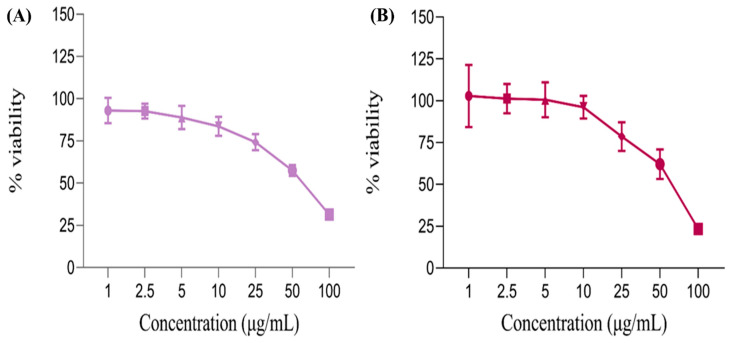
The linear graph shows the percentage of cell viability of (**A**) empty liposomes (EL) and (**B**) the VAL-LP formulation on HaCat cells after 48 h.

The cytotoxicity (cell viability assay) towards the HaCaT cell was evaluated for the optimized liposome formulations (VAL-LP) and empty liposomes (EL). Cytotoxicity was determined by measuring the optical density with a microplate reader, where the % viability was determined. The effect of the formulations (EL and VAL-LP) on HaCat cells after 48 h of exposure is shown in Figure 9.

The tested formulations showed a concentration-dependent effect on HaCaT cells. Notably, at the highest concentration (100 μg/mL), cell viability was lower compared to other concentrations, indicating that VAL-LP exhibited cytotoxicity at higher concentrations. At low concentrations (1–10 μg/mL), the % viability remained above 95%, showing no cytotoxicity. The IC_50_ value for VAL-LP was 65 μg/mL, representing the concentration of VAL-LP reduced cell viability by 50%. There was an inverse relationship between liposome concentration and cell viability: as the concentration of liposomes increased, cell viability decreased, as confirmed by the reported study [77]. The number of viable cells exposed to a high concentration of VAL-LP (100 μg/mL) was reduced compared to those treated with an equivalent amount of EL. This finding aligned with the reported study, which demonstrated that deoxycholate-mediated liposomes (DOC-LS) showed more cytotoxic effects when compared to conventional liposomes [78]. All values for % cell viability were statistically significant (*p* < 0.05).

### 3.2. Fabrication and Characterization of DMNs

In this study, three different concentration ratios (6:3, 8:4, and 10:5 %*w*/*v*) of SA-HPMC were used to prepare sodium alginate combined with hydroxypropyl methylcellulose-based dissolving microneedles (SA-HPMC-DMNs). The following characteristics of the SA-HPMC-DMNs were evaluated: morphology (needle shape), mechanical strength, penetration ability, and dissolution in rat skin. The optimal SA-HPMC concentration for the DMNs was chosen based on the requirements of sharp needle tips, sufficient mechanical strength, sufficient penetration without fracturing, and rapid dissolving properties. The MNs were morphologically identical, bubble-free, and sharp, showing a successful formation of 36 micro projection needles and 6 × 6 pyramidal needles that were uniformly distributed on the patch.

The DMNs, manufactured with three concentration ratios of SA-HPMC (6:3, 8:4, 10:5 %*w*/*v*), were evaluated for their mechanical strength using the texture analyzer. Several factors influence their strength, including the type and concentration of polymers used for fabrication, the conditions during the production of MNs, and the curing method chosen [79]. The main strategies for maintaining needle integrity during insertion have been to use sharp-tipped needles to reduce the amount of insertion force needed and to maximize mechanical strength [80]. After the 32 N axial load was applied, the SA-HPMC-DMNs were 25.09 (0.17)%, 18.95 (0.71)%, and 5.82 (0.42)% (*p* < 0.05), respectively (Figure S4). When the ratio of SA-HPMC in the DMNs increased from 6:3 to 10:5, there was a smaller % height reduction in the microneedles after mechanical analysis, from 25.09 (0.17)% to 5.82 (0.42)% (*p* < 0.05). Specifically, microneedles with the 6:3 ratio showed less mechanical strength compared to those made with other ratios. These developed MN arrays demonstrated appropriate mechanical strength by withstanding significant compression force. Upon compression, bending at the microneedle tips was visible at the SA-HPMC-DMN arrays. SA-HPMC-DMNs, developed with a 10:5 ratio of SA-HPMC, retained their overall morphology without any breaking, while some tip bending was detected during the analysis.

For evaluating the insertion capability of DMNs, a parafilm with a thickness of 125 μm for each layer was used [10]. Although parafilm is commonly used in the literature for preliminary testing, it has limitations in accurately replicating the actual properties of the skin. The penetration in parafilm layers caused by DMNs with different SA-HPMC concentrations (6:3, 8:4, and 10:5 SA-HPMC %*w*/*v*) are shown in Figure S5. The results demonstrated that SA-HPMC-DMNs fabricated with a 6:3% (*w*/*v*) ratio of SA-HPMC were capable of penetrating three parafilm layers; however, the SA-HPMC-DMNs that were produced with an 8:4% and 10:5% (*w*/*v*) ratio penetrated the fourth parafilm layers, though the number of holes reduced with the 8:4% (*w*/*v*) SA-HPMC concentration. In all DMNs, the hole size decreased as the number of layers increased. Additionally, a subsequent analysis was performed on the percentage of holes produced in each parafilm layer (Figure S6). The SA-HMPC-DMNs successfully penetrated the initial two parafilm layers (with 100% microneedles insertion). However, in the third layer, the insertion profile of the needle decreased by 31 (5.58)%, 17 (3.68)%, and 3 (3.75)% (*p* < 0.05) for the SA-HMPC-DMNs prepared with SA-HMPC concentrations of 6:4%, 8:4%, and 10:5 %*w*/*v*. SA-HPMC-DMNs fabricated with an SA-HPMC concentration of 10:5 %*w*/*v* exhibited 17% less insertion in fourth layer. Therefore, SA-HPMC-DMNs, the DMNs fabricated using 6:4% (*w*/*v*) of SA-HPMC, demonstrated an insertion depth of 375 µm. SA-HPMC-DMNs fabricated using 8:4% and 10:5% (*w*/*v*) of SA-HPMC were penetrated further, reaching 500 µm. SA-HPMC-DMNs containing 10:5% (*w*/*v*) of SA-HPMC produced more holes in the parafilm, suggesting a better insertion depth. Based on these findings, it was determined that 10:5% (*w*/*v*) SA-HPMC-DMNs were preferable, and it was anticipated that they would pierce the SC (skin’s outermost layer) since they possessed sufficient mechanical strength and insertion capabilities.

The dissolution characteristics of the DMNs made with varying SA-HPMC concentration ratios (6:3%, 8:4%, and 10:5% SA-HPMC *w*/*v*) were analyzed by inserting them into rat skin and subsequently measuring the residual microneedle length at different time points (Figure S7). The DMNs composed of 6:3 SA-HPMC showed quick dissolving capabilities, dissolving entirely in 6 min. By contrast, DMNs consisting of the 8:4 and 10:5 %*w*/*v* ratios of SA-HPMC completely dissolved in 8 min. Due to their hydrophilic nature and the presence of hydroxyl and carboxyl groups, HPMC and SA are both good candidates for high water solubility [81,82]. The DMNs composed of a 10:5% ratio of SA-HPMC demonstrated fast dissolution, superior insertion ability, and desirable mechanical strength. As a result, the ratio of the SA-HPMC concentration (10:5 %*w*/*v*) was chosen for further studies on DMNs.

#### 3.2.1. Integration of Optimized VAL-LP Formulation into DMNs and Their Characterizations (VAL-LP-DMNs):

The optimized VAL-LP formulation was incorporated into the optimized concentrations of SA-HPMC-DMNs at a 10:5% (*w*/*v*) ratio (creating VAL-LP-DMNs). Combining liposomes with DMNs offers an effective approach that can potentially enhance drug permeability while simultaneously controlling drug release [83]. To ensure that incorporating liposomes into DMNs did not alter their characteristics, the resulting DMNs were carefully examined and characterized. Further studies on drug content and ex vivo analysis were performed.

##### Morphology of VAL-LP-DMNs

**Figure 10:**
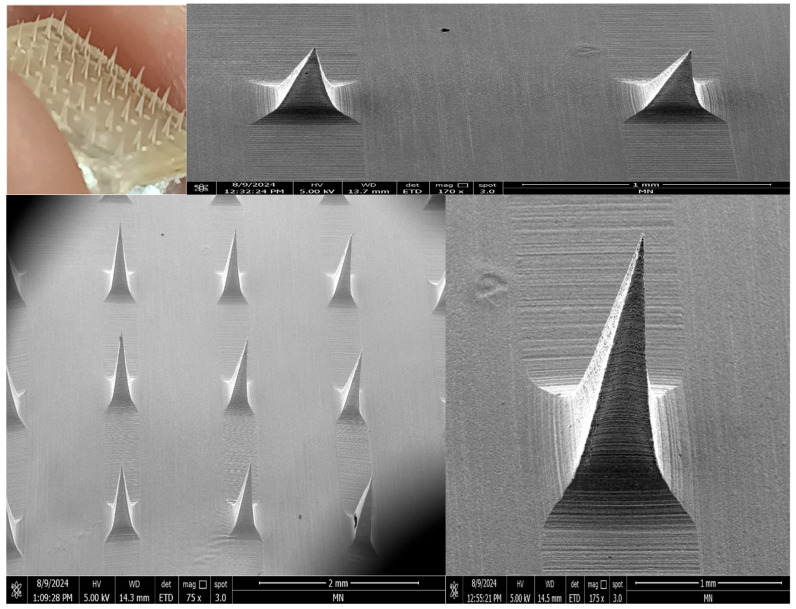
Morphology of VAL-LP-DMNs using digital camera and SEM (magnification 175× and 75×).

The optimized VAL-LP formulation was integrated into SA-HPMC-DMNs, composed of a 10:5% *w*/*v* ratio of SA-HPMC, to enhance the formulation’s handling, permeation, and stability characteristics without delaying the VAL release. The morphological characteristics of optimized VAL-LP-DMNs are shown in Figure 10. DMNs integrated with the VAL-LP formulation exhibiting visible pyramidal micro-projections were observed by scanning electron microscopy (SEM) imaging. These DMNs showed an overall needle length of 802.6 (2.31) µm, a 325.6 (0.30) µm base width, tip-to-tip spacing of 1.59 (0.01) mm, base spacing of 1.07 (0.02) mm, tip diameter of 9.37 (0.01) µm, tip angle of 15.47 (0.21)° and 6 × 6 sharp pyramidal SA-HPMC-DMNs.

##### Mechanical Strength of VAL-LP-DMNs

Drugs entrapped in the DMNs (SA-HPMC-DMNs) may have an impact on the mechanical properties [3]. Thus, employing a texture analyzer, the mechanical strength of the optimized liposome-loaded-DMNs (VAL-LP-DMNs) was further assessed. After compression, the optimized liposome-loaded-DMNs exhibited a reduction in height of 6.87 (0.24)% for the VAL-LP-DMNs compared to their initial height (Figure S8). Additionally, the tips of these optimized liposome-loaded-DMNs (VAL-LP-DMNs) became slightly blunt but showed no significant fractures. These findings suggest that the optimized liposome-loaded-DMNs possess mechanical properties comparable to those of a 10:5 %*w*/*v* SA-HPMC-DMN ratio, demonstrating suitable mechanical strength. These VAL-LP-DMNs showed adequate mechanical strength and even resisted when compression force was applied.

##### Insertion Capability of VAL-LP-DMNs

To assess the penetration ability of the optimized DMN arrays containing VAL-LP, the DMN arrays were inserted into four Parafilm^®^ layers (Figure S9). The created holes (%) in each parafilm layer were analyzed subsequently, as depicted in Figure S10. The insertion profiles of all optimized liposome-loaded DMN formulations were comparable, with a complete insertion (~100% needles) observed in the first two layers, followed by a decrease in the third and fourth layers of parafilm. Additionally, the penetration capability of the liposome-loaded DMN formulations was tested on abdominal rat skin. The DMN array was applied to the skin and subsequently removed from the skin. The dot insertion ratio for the 10:5% (*w*/*v*) VAL-LP-DMNs was 100%. All DMNs produced significant perforations in the rat skin, as shown in Figure S10. These outcomes indicated that optimized VAL-LP-DMNs can penetrate the skin’s stratum corneum effectively. Upon microneedle removal, the skin contracts and the holes created in the skin begin to close due to the natural elasticity of the skin.

##### Dissolution of VAL-LP-DMNs

The dissolution characteristics of the VAL-LP-DMNs formulations were examined at intervals of 2, 4, 6, and 8 min (Figure S11). Over time, each DMN showed a consistent dissolution. Within 6 min, the VAL-LP-DMNs achieved a dissolution rate exceeding 94%. All optimized liposome-loaded DMNs dissolved entirely in 8 min when applied to rat skin, comparable to pure 10:5 %*w*/*v*-SA-HPMC-DMNs. These findings suggested that the optimized liposome-loaded DMN formulations possess self-dissolving qualities and readily dissolve when applied to the skin.

##### SEM and CSLM Analysis of Rat Skin Punctured with VAL-LP-DMNs

**Figure 11:**
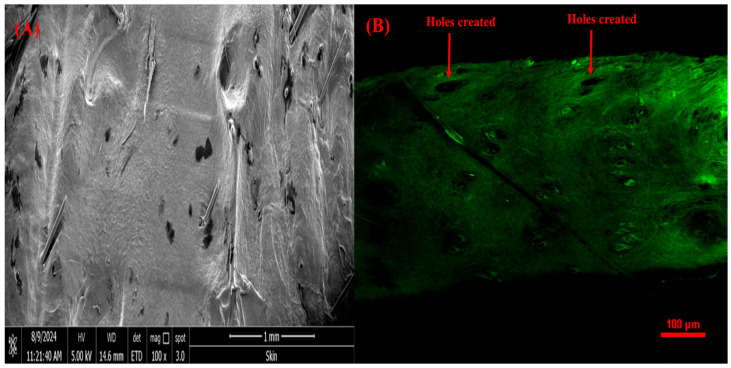
(**A**) SEM image of rat skin treated with optimized VAL-LP-DMNs (100×); (**B**) CSLM image of rat skin treated with VAL-LP-DMNs.

SEM imaging revealed holes at the DMN penetration sites (Figure 11A). Upon microneedle removal, the skin contracts, causing a reduction in the hole’s diameter. Studies have shown that microneedle insertion is limited by skin retraction [43]. CLSM helps to determine if MNs can access the viable epidermis layer. CLSM imaging of the VAL-LP-DMNs revealed holes created in the skin following MN insertion (Figure 11B).

##### Drug Content in Optimized VAL-LP-DMNs

An analysis of the drug content revealed that 46.12 (1.68) µg of VAL loaded into VAL-LP-DMNs resulted in a 38.5% drug loading. The higher viscosity of the final polymer solution containing liposomes may be the reason for the reduced drug quantity in the optimized liposome-loaded DMN formulations. This heightened viscosity might create greater resistance when filling the micromold, affecting drug loading. Similar findings have previously been observed in the reported literature and are more prevalent when liposome-loaded DMNs are involved [10,38].

#### 3.2.2. Ex Vivo Skin Permeation Study of VAL-LP-DMNs

**Figure 12:**
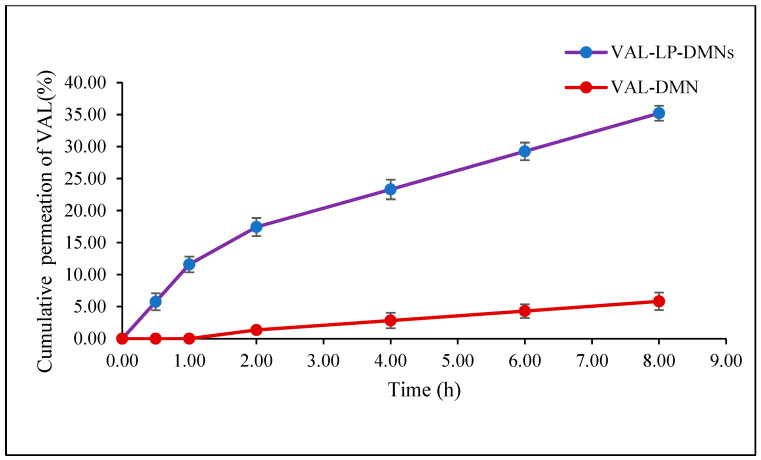
Permeation profiles of VAL-LP-DMNs and VAL-DMNs.

This study assessed the permeation of optimized valsartan-loaded liposome formulations incorporated into DMNs through rat skin over 8 h, as shown in Figure 12. The transdermal flux of VAL-LP-DMNs was 5.36 (0.39) μg/cm^2^/h, while the transdermal flux of VAL-DMNs was 2.89 (0.24) μg/cm^2^/h. The relative increase in ex vivo permeation of the VAL-LP-DMNs to VAL-DMNs was 185% higher. In this study, VAL-DMNs act as controls. The reduced transdermal flux observed in drug-loaded DMNs is associated with the limited water solubility of VAL. The partition coefficient (log P) of VAL 4.5 indicates its lipophilic nature [2]. Consequently, VAL cannot be evenly distributed in water-soluble polymer solutions (SA-HPMC). To address this issue, developing and optimizing an appropriate carrier presents an effective approach to enhance the permeation of VAL. Lipid vesicles offer versatility in carrying both lipophilic and hydrophilic active agents, with adjustable flexibility and deformability based on their composition [84]. The incorporation of VAL as liposome-loaded DMNs significantly improved drug permeation compared to the DMNs loaded with VAL alone, which allows them to penetrate smaller pores.

Poorly water-soluble drugs, such as VAL, dissolve only partially in an aqueous preparation (VAL-DMNs), allowing limited drug permeation through the intact SC [85]. MN-assisted transdermal delivery preserves the advantages of liposomes, such as targeted delivery, controlled release, and drug stabilization, while utilizing the enhanced permeability provided by the pore-forming action of MNs. A recent study by Shen et al. reported enhanced drug permeability of water-soluble drugs through the skin by employing liposome-loaded MNs [83]. Another study also reported enhanced transdermal release with the penetration of liposome-loaded DMNs [86].

Existing valsartan transdermal formulations, including ethosomal gels and nanoethsomes, have shown improved skin permeation and bioavailability compared to oral administration. However, challenges such as formulation stability and skin penetration still need to be addressed [2,22]. Although the ex vivo studies provide valuable insights, they have limitations in predicting clinical performance. These models cannot replicate the full complexity of human skin physiology, such as vascularization and metabolism, which affect bioavailability and systemic absorption. In vivo studies are further needed to confirm the clinical applicability of the formulation.

The current study suggests that liposome-loaded DMNs show promising potential in enhancing drug delivery efficiency through the skin barrier, offering opportunities for non-invasive therapeutic applications.

## 4. Conclusions

An optimized VAL-loaded liposome was integrated into dissolvable microneedles to overcome the barrier properties of the stratum corneum and enhance drug permeation through the skin. The prepared liposomes showed a spherical morphology in nano size; the VAL was solubilized in the liposomes, as confirmed with DSC and FTIR. The in vitro and ex vivo results also indicated better transdermal permeation. The VAL-DMNs results exhibited that a synergistic combination of valsartan-loaded liposome and DMNs significantly improved the permeation of VAL through the skin compared to VAL-LP and VAL-DMNs separately. This enhancement is attributed to the combined effect of liposomes and DMNs, enhancing drug permeation by creating microchannels in the skin. The VAL-LP-DMNs were compared to VAL-LP and VAL-DMNs to assess the added benefits of liposome integration inside DMNs for enhanced drug delivery, skin permeation, and sustained release. The physical characteristics of VAL-LP-DMNs indicated stable and well-structured microneedle artifacts, as evident by SEM. The CSLM image revealed holes created in the skin after DMN insertion, confirming microneedle penetration and the sustained release of VAL.

The limitation for DMNs loaded with nanoparticles is the amount of drug loaded into the needles, which is low compared to transdermal patches. The focus should be on increasing the drug amount in DMNs. Further, an in vivo pharmacokinetic evaluation of these VAL-LP-DMNs to determine the bioavailability of the drug and validate its potential for human trials is required.

## Figures and Tables

**Table 1:** BBD-specified variables for VAL-LP formulations.

Factors	Levels Employed in BBD		
	Low Level (−1)	Medium Level (0)	High Level (+1)
Specified independent variables for BBD			
A = Lipoid P100 (mg)	150	225	300
B = sonication (s)	60	120	180
C = valsartan (mg)	15	22.5	30
Specified dependent variables for BBD			
Y_1_ = particle size of vesicles (nm)			
Y_2_ = ZP (mV)			
Y_3_ = EE (%)			

**Table 2:** Dependent variable values utilized in the BBD for the VAL-LP formulations.

VAL-LP Formulations Design	Independent Variables for BBD			Dependent Variables for BBD		
	A (mg)	B (s)	C (mg)	Y_1_ (nm)	Y_2_ (mV)	Y_3_ (%)
VAL-LP D1	0 [225]	+1 [180]	−1 [15]	288.9 (1.01)	−21.4 (0.35)	99.85 (0.02)
VAL-LP D2	−1 [150]	−1 [60]	0 [22.5]	128.9 (0.01)	−21.6 (0.41)	89.06 (0.05)
VAL-LP D3	0 [225]	−1 [60]	−1 [15]	240.9 (0.91)	−21.3 (0.26)	99.85 (0.07)
VAL-LP D4	0 [225]	+1 [180]	+1 [30]	269.4 (1.60)	−18.0 (0.18)	91.65 (0.01)
VAL-LP D5	+1 [300]	0 [120]	+1 [30]	294.7 (1.01)	−17.2 (0.40)	96.46 (0.03)
VAL-LP D6	−1 [150]	0 [120]	−1 [15]	148.2 (0.58)	−21.9 (0.20)	95.12 (0.08)
VAL-LP D7∗	0 [225]	0 [120]	0 [22.5]	249.4 (0.95)	−23.3 (0.50)	97.91 (0.04)
VAL-LP D8	+1 [300]	+1 [180]	0 [22.5]	246.4 (0.25)	−18.3 (0.25)	98.56 (0.25)
VAL-LP D9	+1 [300]	0 [120]	−1 [15]	226.9 (0.18)	−20.2 (0.75)	98.62 (0.03)
VAL-LP D10∗	0 [225]	0 [120]	0 [22.5]	257.4 (0.58)	−23.9 (0.50)	97.69 (0.11)
VAL-LP D11	0 [225]	−1[60]	+1 [30]	291.6 (0.79)	−20.6 (0.46)	94.56 (0.21)
VAL-LP D12∗	0 [225]	0 [120]	0 [22.5]	244.3 (1.42)	−24.4 (0.75)	97.98 (0.08)
VAL-LP D13	−1 [150]	+1 [180]	0 [22.5]	127.4 (1.01)	−20.9 (0.46)	86.8 (0.05)
VAL-LP D14	+1 [300]	−1 [60]	0 [22.5]	295.4 (0.31)	−17.6 (0.40)	98.56 (0.31)
VAL-LP D15∗	0 [225]	0 [120]	0 [22.5]	252.8 (0.81)	−22.3 (0.23)	96.00 (0.01)
VAL-LP D16	−1 [150]	0 [120]	+1 [30]	132.0 (0.25)	−25.9 (0.75)	84.00 (0.05)
VAL-LP D17∗	0 [225]	0 [120]	0 [22.5]	238.7 (0.50)	−22.9 (0.50)	97.57 (0.08)

∗ Indicates the center point of the design.

**Table 3:** ANOVA analysis for the effect of dependent variables on VAL-LP vesicle size (bold text highlights the statistically not significant, *p* > 0.05 term).

Source	F-Value	*p*-Value	
Model	34.90	<0.0001	Significant
A-Lipoid P100	196.80	<0.0001	
B-Sonication	0.4325	**0.5318**	
C-Valsartan	4.87	**0.0631**	
AB	3.20	**0.1168**	
AC	10.00	0.0159	
BC	7.00	0.0332	
A^2^	87.76	<0.0001	
B^2^	3.23	**0.1153**	
C^2^	3.76	**0.0937**	
Lack of Fit	6.42	0.0521	not significant

**Table 4:** ANOVA analysis for the effect of the dependent variables on VAL-LP zeta potential (bold text highlights not significant *p* > 0.05, term).

Source	F-Value	*p*-Value	
Model	8.27	0.0055	significant
A-lipid	30.18	0.0009	
B-Sonication	0.6825	**0.4360**	
C-Valsartan	0.9956	**0.3516**	
AB	0.3684	**0.563**	
AC	10.15	0.0154	
BC	1.51	**0.2588**	
A^2^	6.85	0.0346	
B^2^	19.70	0.0030	
C^2^	1.52	**0.2579**	
Lack of Fit	2.82	0.1712	not significant

**Table 5:** ANOVA analysis for the effect of dependent variables on VAL-LP EE% (bold text highlights the statically not significant, *p* > 0.05, term).

Source	F-Value	*p*-Value	
Model	43.28	<0.0001	Significant
A-lipid	194.44	<0.0001	
B-Sonication	3.74	**0.0943**	
C-Valsartan	100.58	<0.0001	
AB	1.43	**0.2707**	
AC	22.54	0.0021	
BC	2.37	**0.1674**	
A^2^	59.79	0.0001	
B^2^	1.87	**0.2138**	
C^2^	0.4927	**0.5054**	
Lack of Fit	1.79	0.2888	not significant

**Table 6:** Optimal conditions predicted and experimental values of independent variables for the VAL-LP optimized formulation.

Optimum Independent Variables	Model Coded Levels	Model Actual Levels
A: Lipoid P100 amount (mg)	−0.851	161
B: Sonication time (s)	−0.21	107
C: Amount of VAL (mg)	−0.595	18
Dependent variables	Predicted values	Experimental values
X: Particle size of vesicles (nm)	154.30	150.23 (0.47)
Y: ZP (mV)	−23.21	–23.37 (0.50)
Z: EE%	94.02	94.72 (0.44)

## Data Availability

The original contributions presented in the study are included in the article; further inquiries can be directed to the corresponding author.

## Supplementary Materials

The following supporting information can be downloaded at: https://www.mdpi.com/article/10.3390/pharmaceutics17040483/s1. Table S1: Polydispersity index (PDI) of VAL-LP. Figure S1: Independent variable’s comparative impact on the VAL-LP vesicle size by 3D response surface and contour plot. Figure S2: Independent variable’s comparative impact on zeta potential of VAL-LP by 3D response surface and contour plot. Figure S3: Independent variable’s comparative impact on entrapment efficiency of VAL-LP by 3D response surface and contour plot. Figure S4: Percentage of height reduction in SA-HPMC-DMNs fabricated using three SA-HPMC concentration ratios (6:3, 8:4, and 10:5 %*w*/*v*) after application of the mechanical test. Figure S5: Digital images of parafilm layers showing the insertion of SA-HPMC-DMNs fabricated with three SA-HPMC concentration ratios (6:3, 8:4, and 10:5 %*w*/*v*) (magnification 2×). Figure S6: Percentage of holes and corresponding insertion depth in each layer of parafilm for SA-HPMC-DMNs prepared with three concentration ratios of SA-HPMC: A) 6:3 %, B) 8:4 %, and C) 10:5% (*w*/*v*). Figure S7: Images of dissolution of SA-HPMC-DMNs using three different concentration ratios after insertion into rat skin, (A) 6:3 % SA: HPMC, (B) 8:4 % SA: HPMC, and (C) 10:5% (*w*/*v*) SA: HPMC (magnification 4×). Figure S8: Percentage of height reduction in optimized liposome-loaded-DMNs following mechanical test. Figure S9: Digital images of parafilm layers showing the insertion of optimized VAL-LP-DMNs (magnification 2×). Figure S10: (A) Percentage of holes and corresponding insertion depth in each layer of parafilm for optimized VAL-LP-DMNs; (B) digital image of rat skin surface before and after penetration of optimized VAL-LP-DMNs (magnification 2×). Figure S11: Images of dissolution of optimized VAL-LP-DMNs after insertion into rat skin (magnification 4×).
