# Correction: Khalid et al. Development of Rapidly Dissolving Microneedles Integrated with Valsartan-Loaded Nanoliposomes for Transdermal Drug Delivery: In Vitro and Ex Vivo Evaluation. *Pharmaceutics* 2025, *17*, 483

**DOI:** 10.3390/pharmaceutics17081001

**Published:** 2025-07-31

**Authors:** Ramsha Khalid, Syed Mahmood, Zarif Mohamed Sofian, Zamri Chik, Yi Ge

**Affiliations:** 1 Department of Pharmaceutical Technology, Faculty of Pharmacy, Universiti Malaya, Kuala Lumpur 50603, Malaysia; ramshaamaheen@gmail.com (R.K.); ms_zarif@um.edu.my (Z.M.S.); 2 Universiti Malaya-Research Centre for Biopharmaceuticals and Advanced Therapeutics (UBAT), Department of Pharmacology, Faculty of Medicine, Universiti Malaya, Kuala Lumpur 50603, Malaysia; zamrichik@ummc.edu.my; 3 Centre of Advanced Materials (CAM), Faculty of Engineering, Universiti Malaya, Kuala Lumpur 50603, Malaysia; 4 School of Pharmacy, Queen’s University Belfast, Belfast BT9 7BL, UK

## Figure Legend

In the original publication [1], there was a mistake in the legend for Figure S3. The original Figure S3 displayed a 3D response surface and contour plot related to zeta potential, but it was intended to show the impact of variables on the entrapment efficiency of VAL-LP (Supplementary Material). The correct legend appears below.

**Figure S3.** Independent variable’s comparative impact on entrapment efficiency of VAL-LP by 3D response surface and contour plot.

## Error in Figure

In the original publication [1], there was a mistake in Figure S9. The subfigure labeled “FD-LIPO-DMNs” was mistakenly included in Figure S9. This subfigure does not represent data relevant to the current study and was included by mistake (Supplementary Material). The correct Figure S9 appears below.

**Figure S9:**
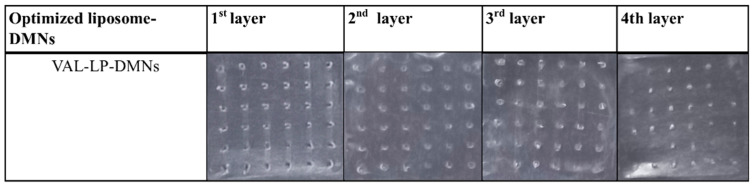
Digital images of parafilm layers showing the insertion of optimized VAL-LP-DMNs (magnification 2×).

There was a mistake in Figure S3 as published. The original Figure S3 displayed a 3D response surface and contour plot related to zeta potential, but it was intended to show the impact of variables on the entrapment efficiency of VAL-LP (Supplementary Material). The corrected Figure S3 appears below. 

**Figure S3:**
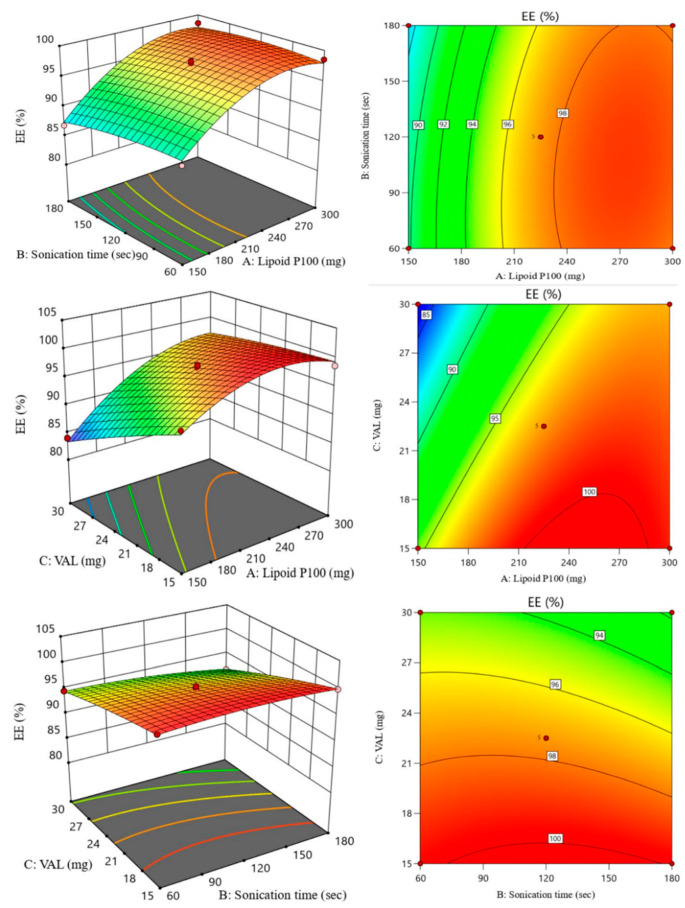
Independent variable’s comparative impact on entrapment efficiency of VAL-LP by 3D response surface and contour plot.

The authors state that the scientific conclusions are unaffected. This correction was approved by the Academic Editor. The original publication has also been updated.
