# Supplementary material for: Development of Rapidly Dissolving Microneedles Integrated with Valsartan-Loaded Nanoliposomes for Transdermal Drug Delivery: In Vitro and Ex Vivo Evaluation
Source: Pharmaceutics. 2025 Apr 7;17(4):483. doi: 10.3390/pharmaceutics17040483 (PMC12030157; doi:10.3390/pharmaceutics17040483)
Supplement: Supplementary file 1 [file pharmaceutics-17-00483-s001.zip › pharmaceutics-3546429-supplementary.pdf]

Supplementary Material:

The PDI was measured for all VAL-LP formulations, as illustrated in Table S1. Concerning the PDI, a value of 0.3 or lower is considered suitable, indicating a homogeneous population of lipid vesicles.

Table S1. Polydispersity index (PDI) of VAL-LP.

**Formulation design**	**PDI**
VAL-LP D1	0.361 (0.01)
VAL-LP D2	0.276 (0.01)
VAL-LP D3	0.279 (0.04)
VAL-LP D4	0.255 (0.01)
VAL-LP D5	0.272 (0.02)
VAL-LP D6	0.370 (0.01)
VAL-LP D7*	0.270 (0.01)
VAL-LP D8	0.395 (0.03)
VAL-LP D9	0.14 (0.02)
VAL-LP D10*	0.250 (0.01)
VAL-LP D11	0.269 (0.01)
VAL-LP D12*	0.401 (0.03)
VAL-LP D13	0.290 (0.01)
VAL-LP D14	0.280 (0.02)
VAL-LP D15*	0.287 (0.01)
VAL-LP D16	0.395 (0.02)
VAL-LP D17*	0.261 (0.01)

* Indicates the center point of the design.

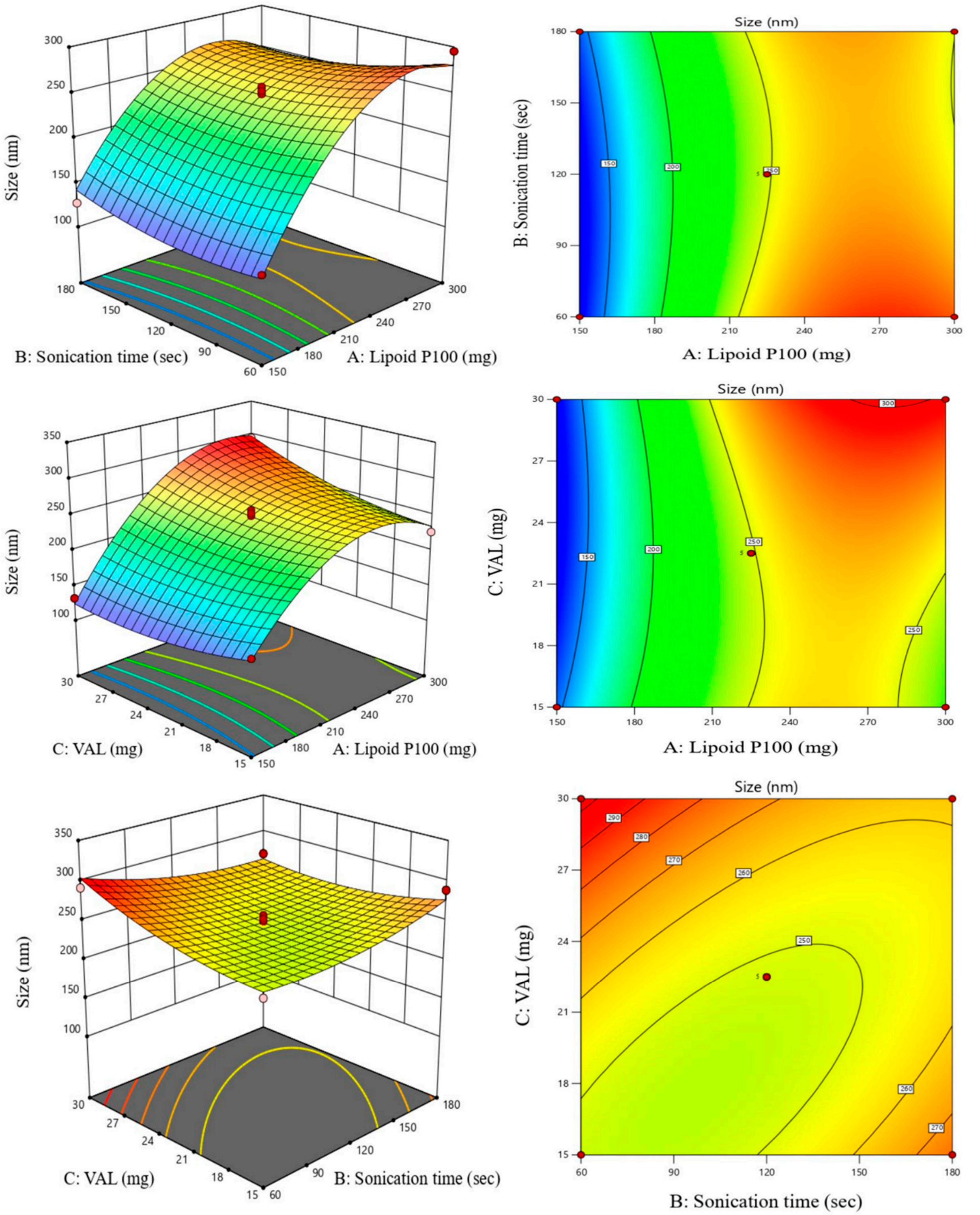

Figure S1. Independent variable's comparative impact on the VAL-LP vesicle size by 3D response surface and contour plot.

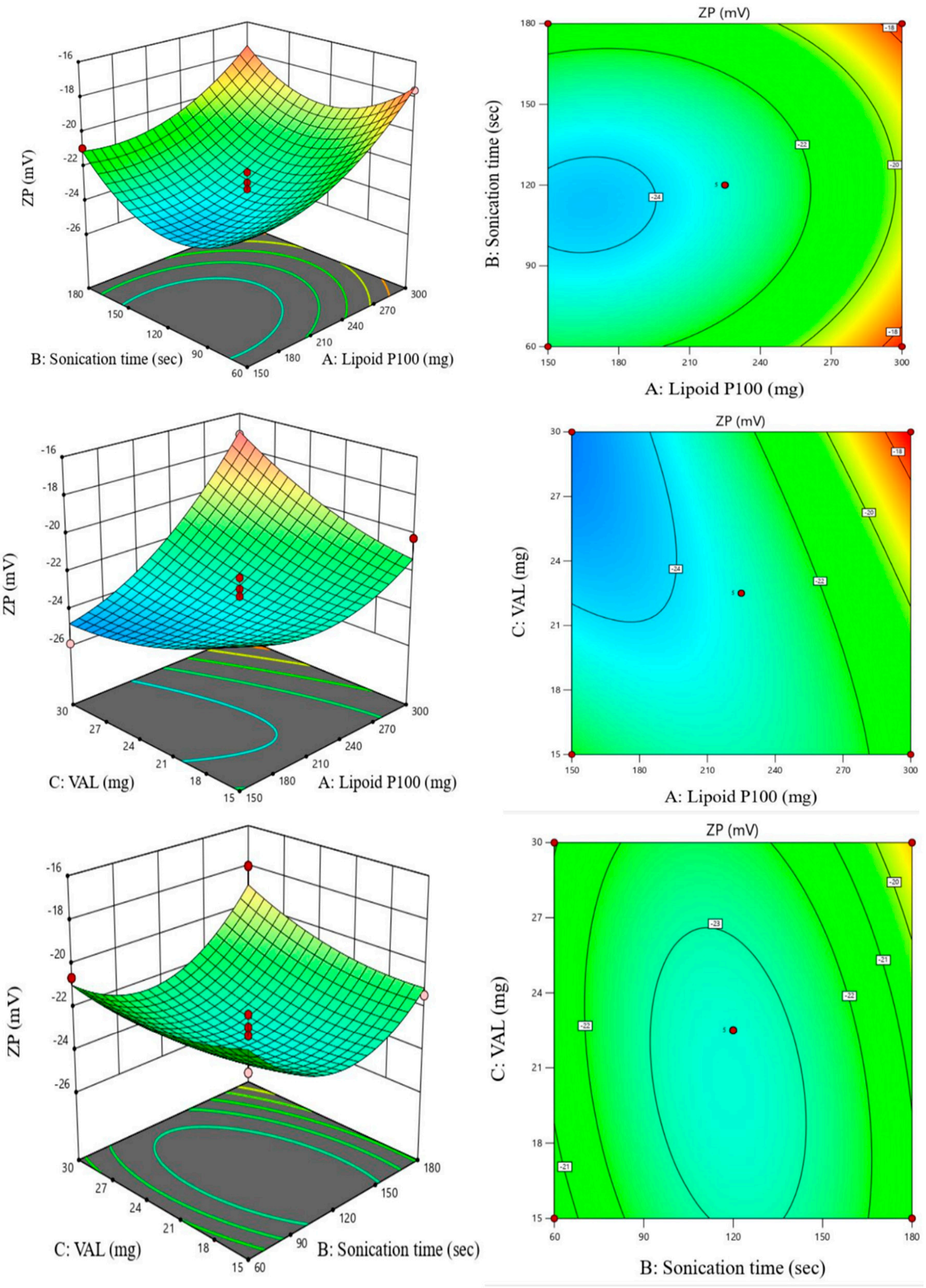

Figure S2. Independent variable's comparative impact on zeta potential of VAL-LP by 3D response surface and contour plot.

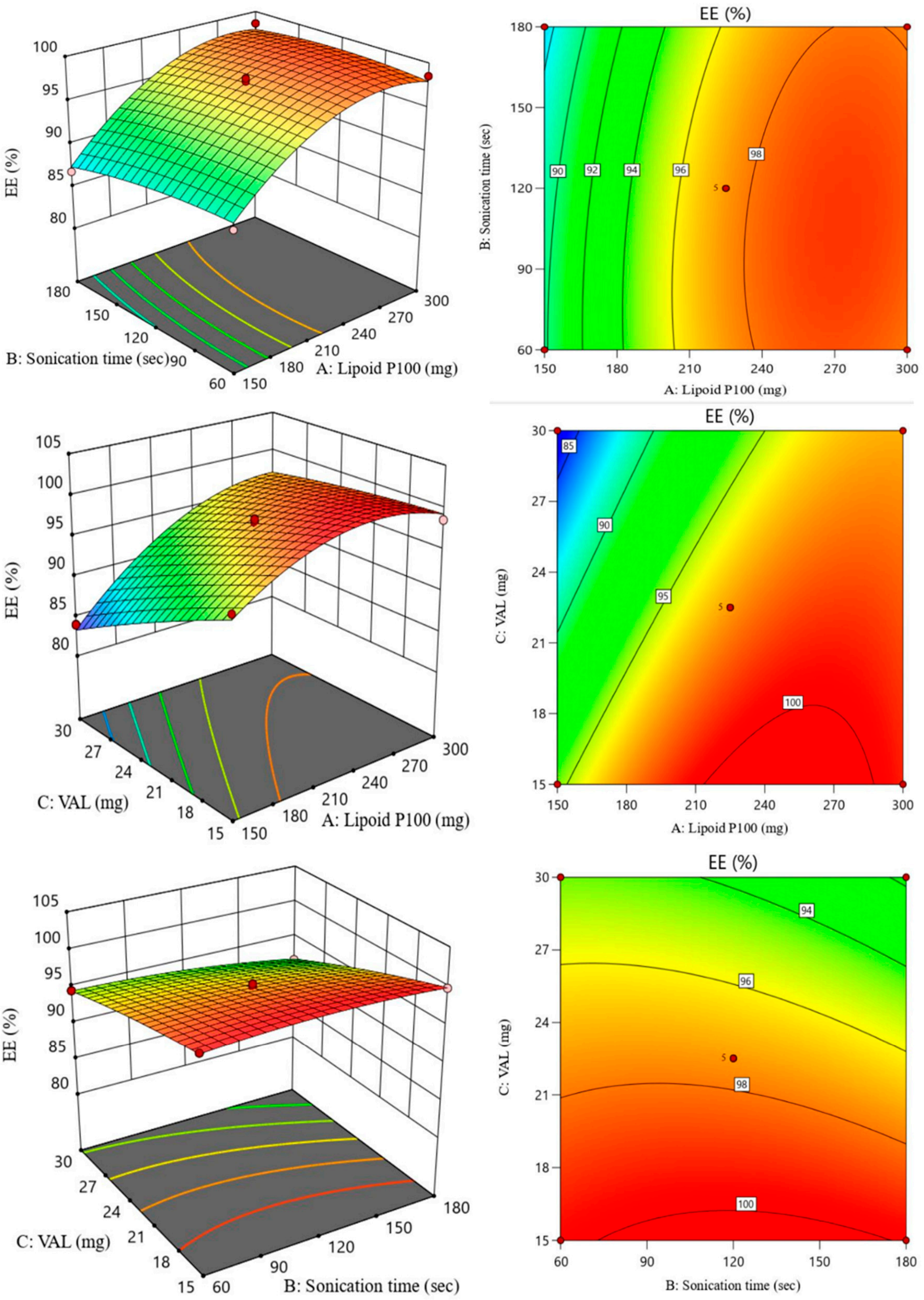

Figure S3. Independent variable's comparative impact on entrapment efficiency of VAL-LP by 3D response surface and contour plot.

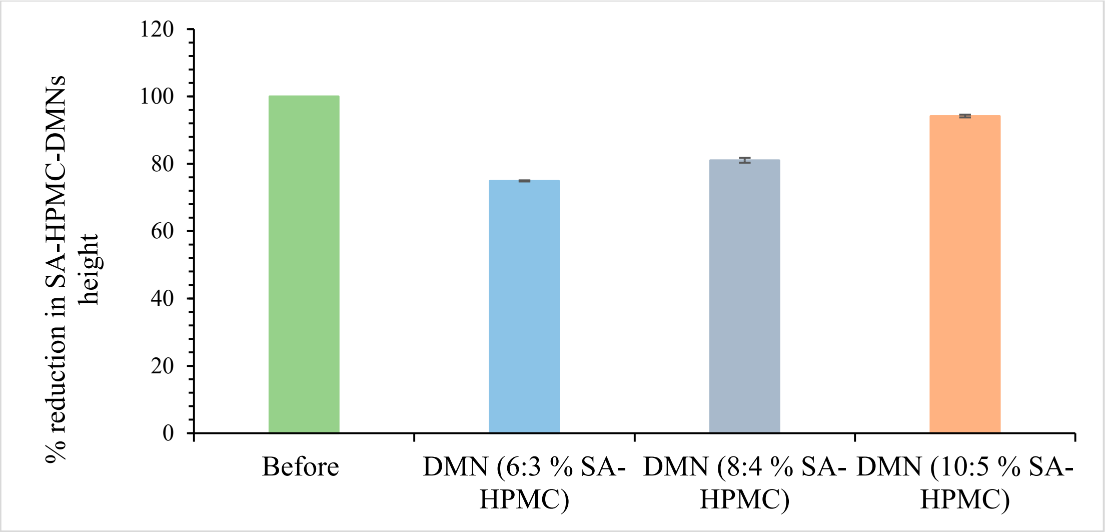

Figure S4. Percentage of height reduction in SA-HPMC-DMNs fabricated using three SA-HPMC concentration ratios (6:3, 8:4, and 10:5 % w/v), after application of mechanical test.

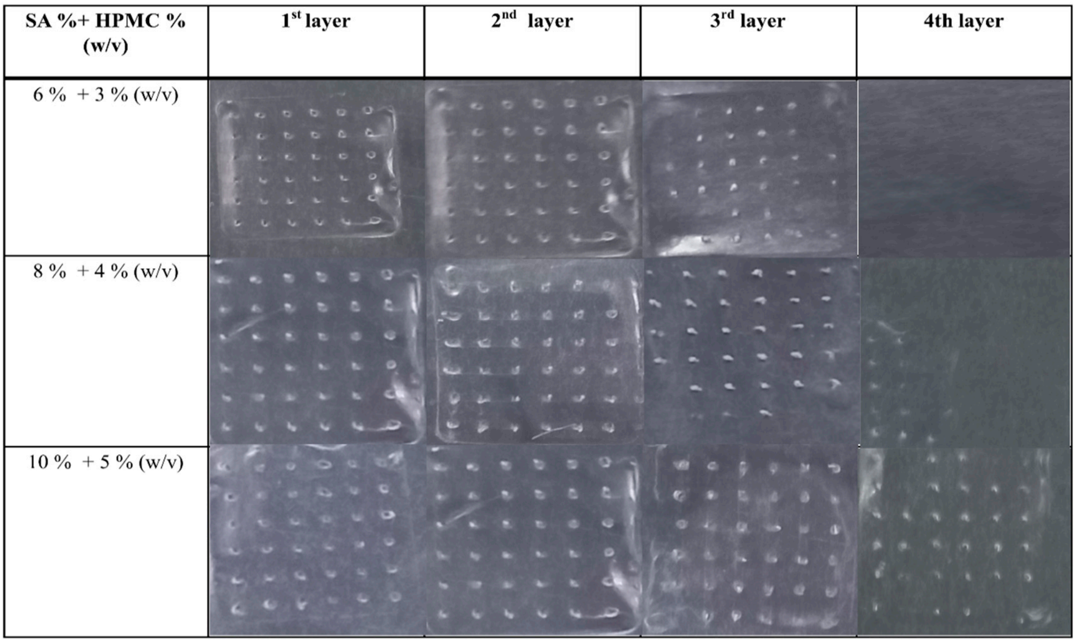

Figure S5. Digital images of parafilm layers showing insertion of SA-HPMC-DMNs fabricated with three SA-HPMC concentration ratios (6:3, 8:4, and 10:5 % w/v) (magnification 2×).

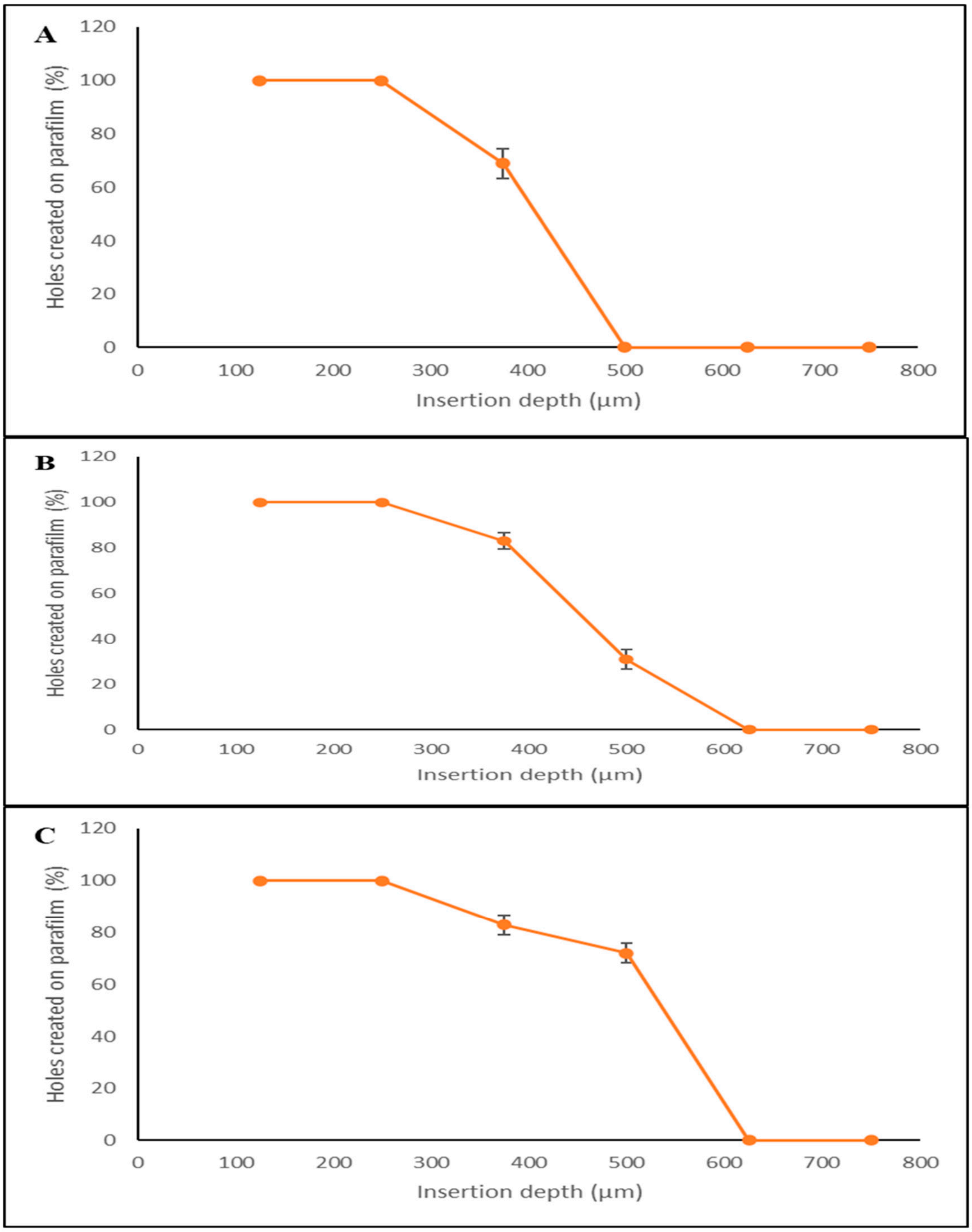

Figure S6. Percentage of holes and corresponding insertion depth in each layer of parafilm for SA-HPMC-DMNs prepared with three concentration ratios of SA-HPMC: A) 6:3 %, B) 8:4 %, and C) 10:5 % (w/v).

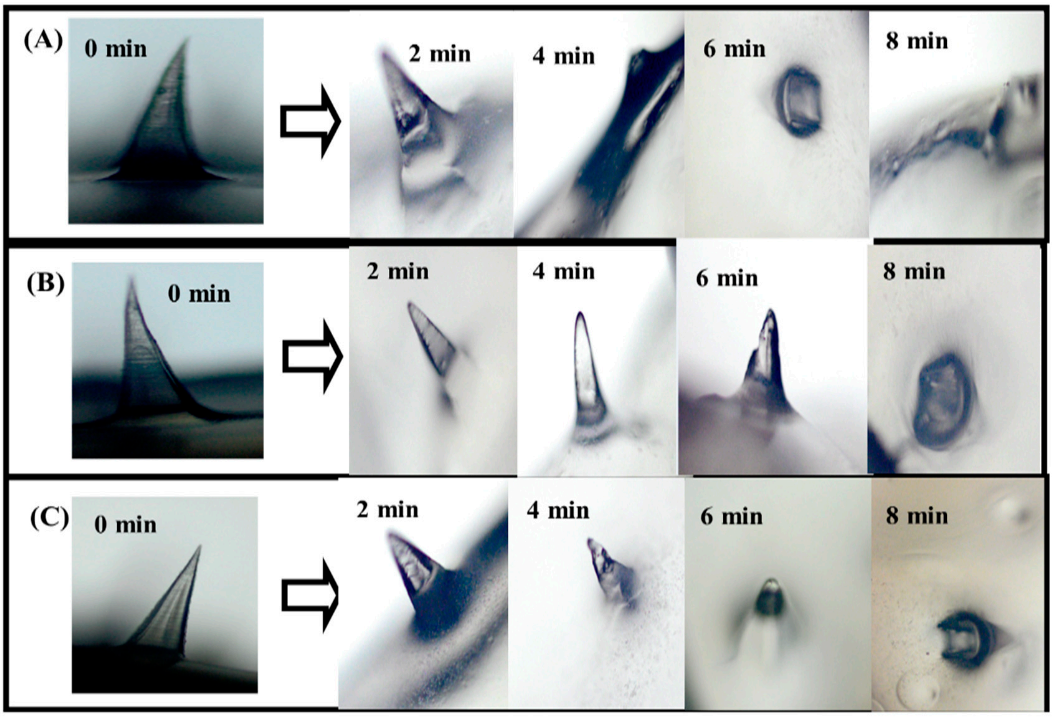

Figure S7. Images of dissolution of SA-HPMC-DMNs using three different concentration ratios after insertion into rat skin, (A) 6:3 % SA: HPMC, (B) 8:4 % SA: HPMC, (C) 10:5 % (w/v) SA: HPMC (magnification 4×).

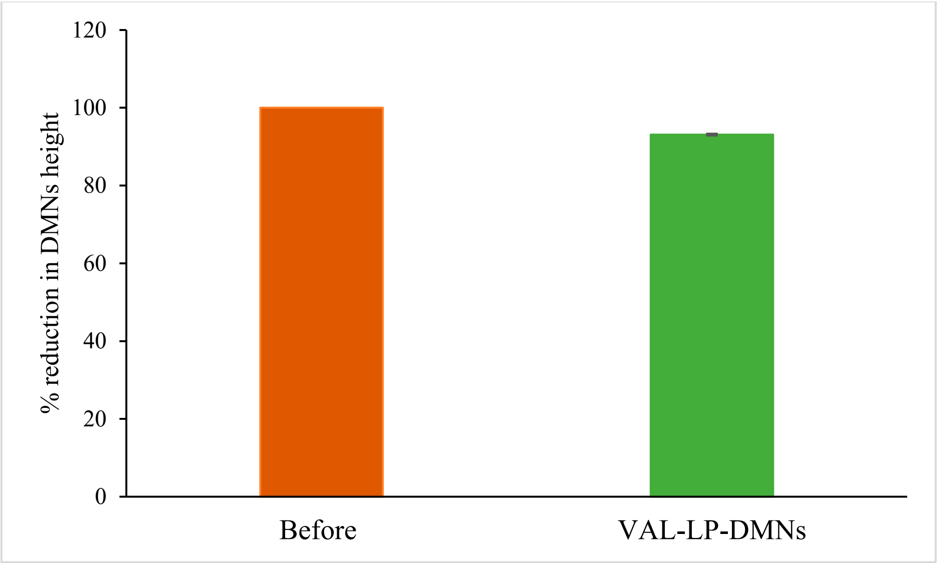

Figure S8. Percentage of height reduction in optimized liposome-loaded-DMNs following mechanical test.

Optimized liposome-DMNs	1st layer	2nd layer	3rd layer	4th layer
VAL-LP-DMNs	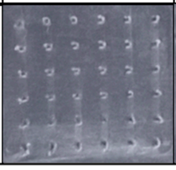	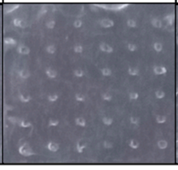	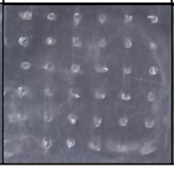	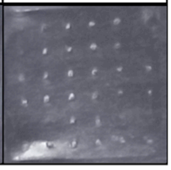

Figure S9. Digital images of parafilm layers showing insertion of optimized VAL-LP-DMNs (magnification 2×).

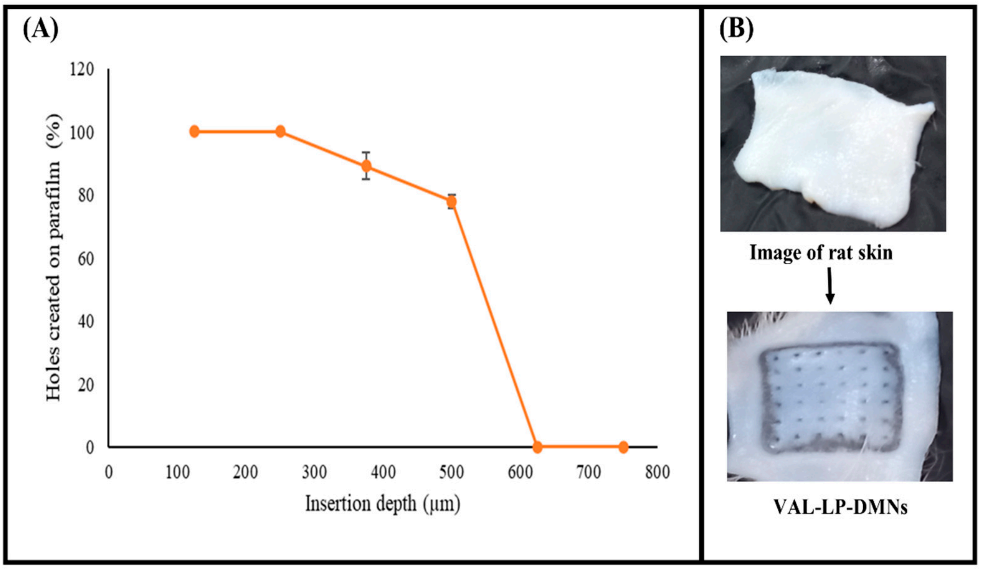

Figure S10. (A) Percentage of holes and corresponding insertion depth in each layer of parafilm for optimized VAL-LP-DMNs, (B) Digital image of rat skin surface before and after penetration of optimized VAL-LP-DMNs (magnification 2×).

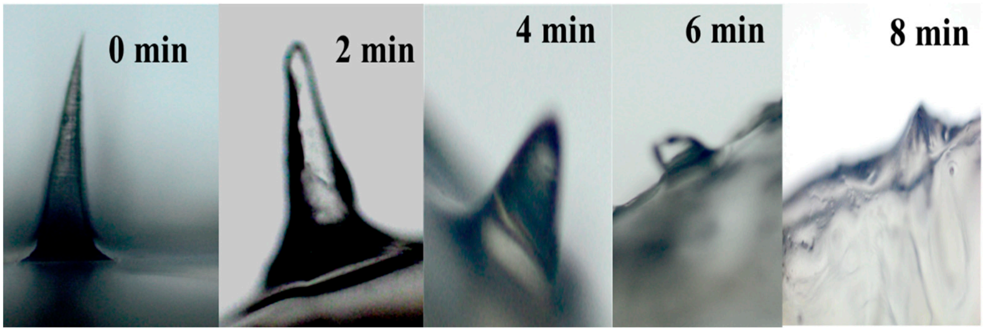

Figure S11. Images of dissolution of optimized VAL-LP-DMNs after insertion into rat skin (magnification 4×).
